# Omics Analyses Uncover Host Networks Defining Virus-Permissive and -Hostile Cellular States

**DOI:** 10.1016/j.mcpro.2025.100966

**Published:** 2025-04-07

**Authors:** Honglin Chen, Philip D. Charles, Quan Gu, Sabrina Liberatori, David L. Robertson, Massimo Palmarini, Sam J. Wilson, Shabaz Mohammed, Alfredo Castello

**Affiliations:** 1 MRC-University of Glasgow Centre for Virus Research, Glasgow, UK; 2 Department of Biochemistry, University of Oxford, Oxford, UK; 3 Big Data Institute, University of Oxford, Oxford, UK; 4 Cambridge Institute of Therapeutic Immunol & Infect Disease, Jeffrey Cheah Biomedical Centre, Cambridge, UK; 5 The Rosalind Franklin Institute, Oxfordshire, UK; 6 Department of Chemistry, University of Oxford, Oxford, UK

**Keywords:** innate immunity, virus, permissiveness, multi-omics, phosphoproteome

## Abstract

The capacity of host cells to sustain or restrict virus infection is influenced by their proteome. Understanding the compendium of proteins defining cellular permissiveness is key to many questions in fundamental virology. Here, we apply a multi-omic approach to determine the proteins that are associated with highly permissive, intermediate, and hostile cellular states. We observed two groups of differentially regulated genes: (i) with robust changes in mRNA and protein levels and (ii) with protein/RNA discordances. While many of the latter are classified as interferon-stimulated genes (ISGs), most exhibit no antiviral effects in overexpression screens. This suggests that IFN-dependent protein changes can be better indicators of antiviral function than mRNA levels. Phosphoproteomics revealed an additional regulatory layer involving non-signaling proteins with altered phosphorylation. Indeed, we confirmed that several permissiveness-associated proteins with changes in abundance or phosphorylation regulate infection fitness. Altogether, our study provides a comprehensive and systematic map of the cellular alterations driving virus susceptibility.

## Highlights

• We provide a multiomics resource to study host determinants of virus permissiveness.
• HEK293 and more permissive HEK293T cells show proteome-wide differential expressions.
• Transcriptome variations cannot fully explain proteome changes in innate immunity.
• Integration of our omics data reveals novel host factors against SINV and HIV-1.

## Graphical Abstract

**Figure:**
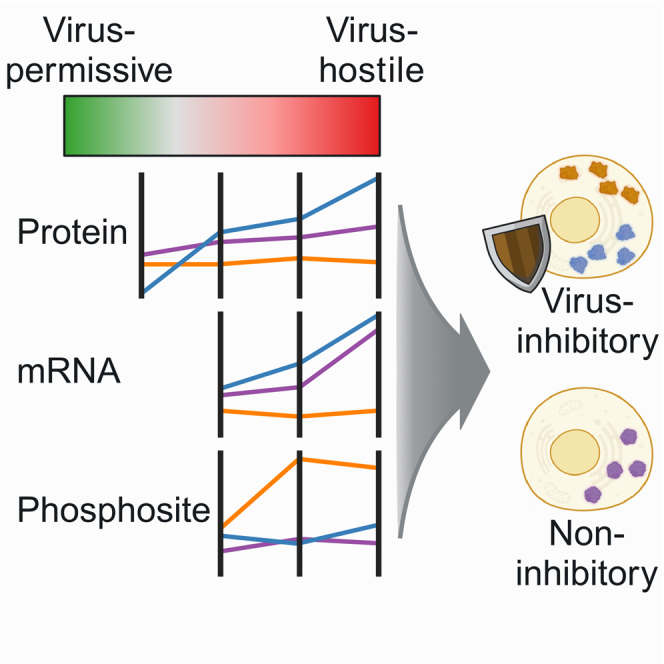

## In Brief

We apply a multiomic approach to determine the proteins that are associated with highly permissive, intermediate, and hostile cellular states. We observed two groups of differentially regulated genes: (i) with robust changes in mRNA and protein levels, and (ii) with protein/RNA discordances. Whereas many of the latter are classified as interferon-stimulated genes (ISGs), most exhibit no antiviral effects in overexpression screens. Phosphoproteomics revealed an additional regulatory layer. Indeed, we confirmed that several permissiveness-associated proteins with changes in abundance or phosphorylation regulate infection fitness.

The limited size of viral genomes renders viruses dependent on host cells; they often provide the molecular machinery required for virus proliferation and spread. Cellular molecules required for virus infection are globally referred to as “dependency” factors (1, 2, 3, 4). These include a wide range of proteins, including, among others, the receptor and co-receptors required for virus entry (5, 6), the cytoskeleton to enable the transport of viral components across the cell (7, 8), cellular co-factors that aid viral replication (9), the translation apparatus necessary for viral protein synthesis (10, 11), the central metabolism to supply these anabolic processes with nucleotides, amino acids and energy (12, 13), the glycosylation required to shield the viral glycoproteins (14), and the ESCRT (endosomal sorting complexes required for transport) to release of the viral particles (15, 16).

On the other hand, almost all human cell types respond to virus infection by initiating immune responses, which begin with the recognition of pathogen-associated molecular patterns (PAMPs) by cellular sensor proteins (17). PAMPs include a wide range of signatures including double-stranded (ds)DNA and dsRNA, 5′-triphosphate, or unmethylated caps at the end of the RNA molecules (18, 19). Sensing of PAMPs triggers signal transduction, which drives the expression and secretion of interferon (IFN). IFN is a family of cytokines consisting of type I, II, and III that initiate the JAK-STAT signaling pathway upon binding to membrane receptors (20). Upon binding to its receptor, IFN triggers the expression of interferon-stimulated genes (ISGs) that facilitate virus sensing and activate mechanisms to antagonize viral replication (21, 22). One example “effector” ISGs include the OAS-RNaseL axis: upon binding to viral RNA, the 2′-5′-oligoadenylate synthase (OAS) proteins initiate the synthesis of 2′-5′-oligoadenylate, which binds to and activates the endoribonuclease RNaseL to induce viral RNA degradation (23, 24).

Permissiveness of the host cell to virus infection is collectively shaped by both host dependency and restriction factors; however, the genes and derived proteins discriminating permissive and hostile cell conditions remain under investigation. As an example, Human embryonic kidney 293 (HEK293) and its derivative HEK293T cells have distinct abilities to sustain virus infection despite their close lineage. This is evidenced by studies showing that HEK293T cells can produce higher titer of murine stem cell virus and human immunodeficiency virus type 1 (HIV-1) (25), and HIV-1-based lentivirus particles (26, 27). HEK293T also produces a higher level of infective adenovirus particles (28, 29, 30), Epstein-Barr virus (31), and Influenza A virus under co-culture conditions (32), and a higher capacity to sustain SARS-CoV-2 replication (33, 34). HEK293T originates from stable transfection of HEK293 with Simian virus 40 large tumor antigen (SV40-LT) (35); however, its different level of permissiveness to viruses cannot be solely attributed to the expression of SV40-LT, because its depletion has a limited effect on virus yield (27, 30), and SV40-LT has multifaceted impacts on innate immunity (36, 37, 38, 39). SLF11 is more abundant in HEK293 than in HEK293T cells, and it was proposed to inhibit HIV-1 by controlling the host aminoacyl-tRNA pool (25). However, SLF11 cannot fully explain the differential ability of HEK293 and HEK293T to sustain HIV-1 infection, and this host factor does not regulate other viruses (25). The genetic variations revealed by a large-scale genome sequencing study comparing HEK cell lines also fail to explain the distinct permissiveness of HEK293 and HEK293T (40). The determinants of virus permissiveness between these closely related cell lines thus remain largely unknown.

HEK293 and HEK293T cell lines provide a model system to study determinants of virus permissiveness due to their differential capacity to sustain infection despite having closely related genomes (41). In this study, we employed an integrated omics analysis to uncover the scope of cellular proteins that define virus permissiveness across multiple cellular states, including the HEK293T cells (highly permissive), steady-state HEK293 cells (intermediate), and HEK293 upon stimulation of IFN-α (hostile). Our results revealed a subset of antiviral factors that are globally depleted in HEK293T cells while anabolic pathways are upregulated, creating an ideal environment for virus replication. In addition, our transcript- and proteomics data depicted a temporal gene expression regulation in the IFN-α response. This analysis pinpointed a group of antiviral factors that is robustly and reproducibly upregulated in response to IFN-α. Moreover, we observe another group of genes that despite being upregulated at the RNA level, do not show changes at the protein level, which suggests IFN-α-induced transcriptional noise with limited effects at regulating infection. Moreover, our phosphoproteomic analysis uncovered extensive modulation of phosphorylation among non-signaling proteins in response to IFN-α. Through integrated analysis of omics data, we identified novel regulators of virus infection and provided experimental validations on their regulatory roles against Sindbis virus (SINV) and HIV-1.

## Experimental Procedures

### Experimental Design and Statistical Rationale

All proteomics, phosphoproteomics, and transcriptomics experiments were performed in biological triplicates. Proteomics and phosphoproteomics samples were SILAC labeled and mixed with label swap prior to LC-MSMS to evenly distribute isotope channels across experimental conditions. Only entries with at least three quantified data points in at least one condition were considered in quantitative analysis, entries with two quantified data points in at least one condition were considered in semi-quantitative analysis. This filtering criteria was chosen to increase statistical stringency. Statistical analysis was performed with empirical Bayes moderated *t* test in R (v.4.3.2, R Core Team). *p* values derived from t-tests were adjusted for multiple-testing with the Benjamini-Hochberg method to generate FDR.

All plate reader-based fluorescence recording assays were performed in technical triplicates on each plate and biological triplicates on separate plates. Control groups were uninduced and uninfected cells, readouts from these groups were subtracted from experimental groups to reduce noise, and signals in experimental groups were scaled to 0∼100 based on signal at 24/48 hpi in uninduced and SINV/HIV-1 infected cells, respectively. Statistical analysis was performed with a two-tailed unpaired *t* test in R.

### Cell Culture

The following human cell lines are available commercially available: HEK293 cell line (ECACC, #85120602), HEK293 Flp-In TRex cell line (Thermo Fisher Scientific, #R78007). HEK293T cells were kindly provided by Prof. Jan Rehwinkel (University of Oxford, UK). HEK293 Flp-In TRex inducible expression cells expressing proteins of interest were generated with Flp-In TRex Core Kit (Thermo Fisher Scientific, #K6500-01) according to manufacturer’s protocol, plasmids used for inducible cell line generation are described in following sections.

Cells were cultured in DMEM supplemented with 10% fetal bovine serum (FBS) and 1× penicillin/streptomycin (Sigma Aldrich, #P4458) and the following specific antibiotics: 100 μg/ml Zeocin (Thermo Fisher Scientific, #R25001) and 7.5 μg/ml Blasticidin S Hydrochloride (Cambridge Bioscience, #B001-100 mg) for HEK293 Flp-In TRex; 350 μg/ml Hygromycin B (Cambridge Bioscience, #H011-20 ml) and 7.5 μg/ml Blasticidin for HEK293 Flp-In TRex inducible expression cells. All cells were cultured in a humidified incubator at 37 °C with 5% CO_2_. Culture with interferon-alpha (IFN-α) stimulation was performed by supplying IFN-α stocks into culture media at indicated concentration and time period prior to harvest. IFN-α stocks were obtained by dissolving commercial IFN-α (PBL assay science, #11100-1) with 0.1% BSA in water and stored at −80 °C.

### Cell culture in SILAC Media

Cells were cultured in arginine and lysine depleted DMEM (Thermo Scientific, #10107883) supplied with 10% dialyzed FBS (Silantes GmbH, #281000900), 1× penicillin/streptomycin, and isotope labeled arginine and lysine (Silantes GmbH amino acids: L-Arginine 13C,15N #201604102; L-Arginine 13C #201204102; L-Lysine 13C,15N #211604102; 4.4.5.5.-D4-L-Lysine #211104113). Cells were cultured in corresponding SILAC media for at least 5 more passages before experiments. Isotope incorporation rates over 99% were confirmed by mass spectrometry using whole cell lysates.

### Viruses

SINV_mCherry_ suspension was generated with plasmid pT7-SVmCherry as described in our previous study (42). Pseudotyped HIV-_1Nef-mCherry_ and HIV-_1Gag-mCherry_ were generated by co-transfection of HEK293T cells with pNL4-3. R-E- derived plasmids and a plasmid encoding the vesicular stomatitis virus glycoprotein (pHEF-VSVG, NIH AIDS Reagent Program, #4693) as described in our previous study (42).

### Plasmids

Plasmids for producing inducible cell lines were generated by conventional cloning methods and gene synthesize: coding sequences of DTX3L and UBE2L6 were amplified from cDNA of IFN-α stimulated HEK293 using specific primers (supplemental Table S6), coding sequences of BTBD2, CMPK2, EPSTI1, FRMD5, HERC6, HNRNPD, LGALS3BP, MTRF1, NCOA4, POLDIP3, THAP10, UBE2J2, USP41 were generated by gene synthesis through Bio Basics Inc (supplemental Table S6). Cloned and synthesized sequences were flanked with BamHI/HindIII/KpnI at 5′ and NotI at 3′ ends restriction sites and cloned into the vector pcDNA5/FRT/TO with eGFP preceded or followed by a Gly-Ser linker sequence (GGSGGSGG).

### Fluorescence-Based Virus Fitness Assay

Cells were seeded on a 96-well microplate (flat-bottom, Greiner Bio-One, #655986) at 60,000 cells per well, in complete DMEM (lack phenol-red) supplied with 5% FBS, 1 mM sodium pyruvate, 1× penicillin/streptomycin. For IFN-α stimulation assays, IFN-α was supplemented to each well at specified concentrations at least 4 h after cell seeding. For Flp-In TRex cell lines, 1 μg/ml doxycycline was supplied in culture media during cell seeding. Infection of SINV_mCherry_, HIV-1_Gag-mCherry,_ or HIV-1_Nef-mCherry_ was performed at least 20 h after cell seeding with complete DMEM (lack phenol-red) containing 2.5% FBS, 1 mM sodium pyruvate, 1× penicillin/streptomycin, and virus at specified MOI. Cells were incubated at 37 °C and 5% CO_2_ in a CLARIOstar fluorescence plate reader (BMG Labtech) for 24 h (SINV) or 48 h (HIV-1). Fluorescence results were obtained with n ≥ 9 for SINV assays (3 replicates per plate, ≥3 plates) and n ≥ 6 for HIV-1 assays (3 replicates per plate, ≥2 plates).

Fluorescence signals collected from CLARIOstar plate reader were analyzed with R. For analysis of statistical significance, mCherry signals were scaled to 0 to 100 to account for between-plate variations, then determined by *t* test. For analysis of fluorescence signal delay with mCherry-tagged viruses, the average of scaled mCherry signals at each timepoint was calculated for each plate, and the fluorescence signal delay (Δt) at each timepoint on each plate was obtained by finding the earliest timepoint that the average signal in overexpressed cells exceeds that in control cells, these Δt values were then summarized into Δt-over-hpi plots by plotting the average and standard deviation of at each Δt timepoint. Analysis of slopes of the Δt-over-hpi curve was performed with linear regression using R.

### Proteomics Sample Preparation

SILAC samples for HEK293 *versus* HEK293T cells proteomics analysis were cultured on 6-well plate (2 light + 1 heavy samples for HEK293, 2 heavy + 1 light samples for HEK293T); samples for HEK293 mock *versus* IFN-α stimulated cells proteomics and phosphoproteomics analysis were cultured on 150 cm^2^ dishes (1 light + 1 medium + 1 heavy samples for mock, IFN-α 10 min, IFN-α 4 h, and IFN-α 20 h). Cells were lysed on a plate with urea lysis buffer (8 M urea (Sigma, U1250), 100 mM ammonium bicarbonate (AmBic; Sigma, #9830), 10 μl/ml protease inhibitor cocktail (Sigma, P8340) and 2.5 μl/ml phosphatase inhibitor (Sigma, P0044) after gentle washes with cold PBS. Protein concentration in each sample was measured with Pierce 660-nm Protein Assay (Thermo Scientific, #22660) followed by sample mixing with label swap.

For proteomics analysis, combined lysates were reduced and alkylated with the addition of 10 mM Tris(2-carboxyethyl)phosphine hydrochloride (TCEP; Thermo Scientific #77720) and 50 mM 2-Chloroacetamide (CAA; Sigma, #C0267) and in-dark incubation for 30 min. Alkylated samples were diluted to 6 M urea with 25 mM AmBic for LysC protease (Wako, #129-02541) digestion with protein/protease ratio at 40:1 (w/w) at 37 °C for 4 h, followed by dilution to 1 M urea with 25 mM AmBic and 37 °C o/n digestion with trypsin protease (MS grade; Promega, V5280) in protein/protease ratio of 40:1 (w/w). Digestion was quenched with 0.5% (v/v) formic acid (FA; Thermo Scientific, A117–50) and frozen in −80 °C prior to fractionation. Tryptic peptide fractionation was performed with off-line HPLC, loaded with solvent A (10 mM AmBic 2% acetonitrile (ACN; Sigma, #34851) in water, pH 8.3) and separated by a Zorbax 300 Extended-C18 column (2.1 × 150 mm, 3.5 μm) using a linear gradient (length: 100 min, 8% to 60% solvent B (80% ACN in water), flow rate: 200 μl/min). Fractions were collected every 1-min interval between 12 to 92 min of the gradient. The 80 collected fractions were combined at an even interval (first, 21st, 41st, and 61st fractions were combined, and so on), resulting in 20 fractions. Fractions were dried with SpeedVac and stored in −80 °C, and reconstituted with loading buffer (5% DMSO, 5% FA in water) prior to LC-MSMS analysis.

For phosphoproteomics analysis, combined samples were reduced and alkylated with the addition of 10 mM TCEP and 50 mM CAA and in-dark incubation for 30 min. Alkylated samples were purified with methanol-chloroform precipitation by sequential supply of methanol, chloroform, and water in 4:1:3 ratio (v/v) with vortex after each reagent addition, followed by removal of the upper layer and addition of 3 volumes of methanol, then followed by centrifugation in 3000 g for 10 min and removal of all liquid. The remaining samples were air-dried and reconstituted in 8 M urea buffer. Purified alkylated proteins were diluted to 6 M urea with 25 mM AmBic for LysC protease digestion with protein/protease ratio at 60:1 (w/w) at 37 °C for 4 h, followed by dilution to 1 M urea with 25 mM AmBic and 37 °C o/n digestion with trypsin protease in protein/protease ratio of 50:1 (w/w), then quenched with 0.5% (v/v) FA. Fractionation of tryptic peptide was performed with the same method as proteomics samples resulting in 20 fractions, which were dried with Speed Vac and resuspended in 125 μl loading buffer, then combined into 10 fractions at an even interval (first with 11th, second with 12th, etc). These 10 fractions were subjected to phosphopeptide enrichment by Ti-IMAC (Resyn Bioscience, MR-TIM002) according to the manufacturer’s protocol. Final eluates were dried with SpeedVac and stored in in −80 °C prior to LC-MSMS analysis.

### Mass Spectrometry

For proteomics analysis, reconstituted tryptic peptides were analyzed on a nanoUHPLC (Thermo) connected to a Q Exactive mass spectrometer (Thermo Fischer Scientific) through an EASY-Spray nano-electrospray ion source (Thermo Fischer Scientific). The peptides were trapped on a C18 PepMap100 pre-column (300 μm i.d. × 5 mm, 100 Å, Thermo Fisher) using solvent A (0.1% formic acid in water). The peptides were separated on an EASY-spray Acclaim PepMap analytical column (75 μm i.d. × 500 mm, RSLC C18, 2 μm, 100 Å) using a linear gradient (length: 120 min, 8% to 28% solvent B (0.1% formic acid, 5% DMSO in acetonitrile), flow rate: 200 nl/min). The separated peptides were electrosprayed directly into the mass spectrometer operating in a data-dependent mode. Full scan MS spectra were acquired in the Orbitrap (scan range 350–1500 m/z, resolution 70,000, AGC target 3e6, maximum injection time 100 ms). After the MS scans, the 20 most intense peaks were selected for HCD fragmentation at 30% of normalized collision energy. HCD spectra were also acquired in the Orbitrap (resolution 17,500, AGC target 5e4, maximum injection time 120 ms).

For phosphoproteomics analysis, Ti-IMAC enriched phosphopeptides were reconstituted in a loading buffer and analyzed on two instruments. One-half of reconstituted phosphopeptides were analyzed on a nanoUHPLC (Thermo) connected to a Q Exactive mass spectrometer (Thermo Fischer Scientific) through an EASY-Spray nano-electrospray ion source (Thermo Fischer Scientific). The peptides were trapped on a C18 PepMap100 pre-column (300 μm i.d. × 5 mm, 100 Å, Thermo Fisher) using solvent A (0.1% formic acid in water). The peptides were separated on an EASY-spray Acclaim PepMap analytical column (75 μm i.d. × 500 mm, RSLC C18, 2 μm, 100 Å) using a linear gradient (length: 120 min, 8% to 28% solvent B (0.1% formic acid, 5% DMSO in acetonitrile), flow rate: 200 nl/min). The separated peptides were electrosprayed directly into the mass spectrometer operating in a data-dependent mode. Full scan MS spectra were acquired in the Orbitrap (scan range 350–1500 m/z, resolution 70,000, AGC target 3e6, maximum injection time 50 ms). After the MS scans, the 20 most intense peaks were selected for HCD fragmentation at 30% of normalized collision energy. HCD spectra were also acquired in the Orbitrap (resolution 17,500, AGC target 5e4, maximum injection time 120 ms). The other half of the phosphopeptide samples were analyzed on an EASY-nLC 1000 System (Thermo) connected to an Orbitrap Elite Hybrid mass spectrometer (Thermo Fischer Scientific) through an EASY-Spray nano-electrospray ion source (Thermo Fischer Scientific). The peptides were trapped on a C18 PepMap100 pre-column (300 μm i.d. × 5 mm, 100 Å, Thermo Fisher) using solvent A (0.1% formic acid in water). The peptides were separated on an EASY-spray Acclaim PepMap analytical column (75 μm i.d. × 500 mm, RSLC C18, 2 μm, 100 Å) using a linear gradient (length: 60 min, 8% to 28% solvent B (0.1% formic acid, 5% DMSO in acetonitrile), flow rate: 200 nl/min). The separated peptides were electrosprayed directly into the mass spectrometer operating in a data-dependent mode. Full scan MS spectra were acquired in the Orbitrap (scan range 350–1500 m/z, resolution 70,000, AGC target 1e6, maximum injection time 100 ms). After the MS scans, the 20 most intense peaks were selected for CID fragmentation at 35% normalized collision energy. CID spectra were acquired in the LTQ mass analyzer (resolution 7500, AGC target 5e3, maximum injection time 100 ms).

Protein identification and quantification were performed using the Andromeda search engine implemented in MaxQuant (1.6.3.4). Peptides were searched Human Uniprot database (Uniprot_id: UP000005640, downloaded Nov 2016). Multiplicity was set to 2 for HEK293-*versus*-HEK293T proteomics analysis, specifying Arg10/Lys8 for heavy labels; while multiplicity was set to 3 for HEK293-*versus*-IFN-stimulated HEK293 proteomics and phosphoproteomics analysis, specifying Arg6/Lys4 for medium labels and Arg10/Lys8 for heavy labels. False discovery rate (FDR) was set at 1% for both peptide and protein identification. Phospho(STY) was set as a variable modification for phosphoproteomics analysis, and raw files from two instruments were searched separately. Data files from the same sample were assigned as near-by fractions, and match between runs was turned on to match peak information from near-by fractions. For all searches, the number of miss cleavages permitted was set to 2, Carbamidomethyl (C) was set as fixed modifications, oxidation (M) and Acetyl (protein N-term) was set as variable modification, mass tolerance for precursor ion was set to 20 ppm in the first search and 4.5 ppm in main search, MS/MS mass tolerance was set to 20 ppm for FTMS and 0.5 Da for ITMS. Other options were set as defaults.

### Proteomics Quantitative Analysis

For protein quantification, proteinGroups files from MaxQuant search outputs were used for quantitative analysis. Analysis was performed in R (v.4.3.2, R Core Team). Proteins flagged as decoys or potential contaminants were filtered out. Proteins with at least 3 valid values in at least one condition were subjected to log 2 transformation, normalization, and imputation of missing values. Normalization was performed with vsn method using package *vsn* (43), and imputation was performed by background-level signal using deterministic minimum method (minDet) in package *ImputeLCMD* (44). Principal component analysis (PCA) was performed to evaluate batch effects prior to imputation. Statistical analysis was performed using empirical Bayes moderated *t* test in package *limma* (45). For HEK293-vs-HEK293T proteomics analysis, the replicate number was set as a block variable to account for experimental errors after mixing SILAC samples with label swap (46); for HEK293 mock-vs-IFN-α proteomics analysis, the replicate number was set as block variable and isotope type was modeled as co-variable in design matrix. *p* values were adjusted for multiple testing with Benjamini-Hochberg method.

For phosphosite quantification, Phospho(STY) files from MaxQuant search outputs were used for quantitative analysis. Phosphosites with at least 3 valid values in at least one condition were subjected to log 2 transformation, normalization, and imputation of missing values. Normalization was performed with vsn method using package *vsn*, imputation was performed by background-level signal using deterministic minimum method (minDet) in package *ImputeLCMD*. Principal component analysis (PCA) was performed to evaluate batch effects prior to imputation. Statistical analysis included both moderated *t* test and Z test. Moderated *t* test was performed using package *limma* with a replicate number set as block variable and isotope type modeled as co-variable. Z test was performed by obtaining the Z-score *via* dividing average fold change of each phosphosite by the global standard deviation, then converting Z-scores to *p*-values. All *p* values were adjusted for multiple testing with Benjamini-Hochberg method. For phosphosites that did not meet quantification criteria, sites detected with 2 valid values in one condition and completely missing in the other condition were selected for semi-quantitative analysis by calculating intensity, fold change, and rank among total semi-quantitation sites.

Phosphoproteome data from two instruments was tested separately and these analysis results are available in supplemental Table S4 “test result, OrbitrapElite” and “test result, Q-Exactive”. Test results from two instruments were further combined following these rules: (1), all sites only quantified in one instrument were accepted; (2), for sites quantified in both instruments, accept the result with higher localization probability; (3), if the difference between localization probabilities was less than 0.05, accept the result with higher identification score; (4), if both results scored over 90, accept the result with higher quantile in the dataset. The combined phosphosite quantification result is available in supplemental Table S4 “Combined test result” table.

### RNA Sequencing

HEK293 cells were cultured on 6-well plates in triplicate with IFN-α treatments (mock, IFN-α 4 h, IFN-α 20 h). Total RNA was extracted from cells using Monarch Total RNA Miniprep Kit (NEB, T2010S) following the manufacturer's protocol. Briefly, cells were gently washed with 4 °C PBS and lysed with 600 μl RNA Lysis Buffer provided in the RNA miniprep kit with gentle pipetting. Genomic DNA (gDNA) was removed using gDNA Removal Column with a centrifuge, and flow-throughs were supplied with ethanol and loaded on RNA Extract Columns with a centrifuge. RNA on the column was washed with Washing Buffer, then eluted with 100 μl nuclease-free water. Ribosomal RNAs were removed by rRNA Depletion Kit (NEB, E6310) following the manufacturer’s protocol. Briefly, total RNA was hybridized with rRNA probes in rRNA Depletion Solution, then sequentially digested by RNase H and DNase I at 37 °C for 30 min each. Extracted RNAs were supplied with RiboLock RNase inhibitor (Thermo Scientific, EO0381) and subjected to SuperScript III reverse transcriptase (Invitrogen, #18080093) for cDNA library generation using random hexamer primers. RNase H was added to reverse transcription products to obtain cDNA library for RNA-seq. Libraries were pooled in equimolar concentrations and sequenced using a NextSeq 500 sequencer (Illumina).

RNA-Seq reads quality was assessed using FastQC software (http://www.bioinformatics.babraham.ac.uk/projects/fastqc). 89.1% of the reads generated presented a Q score of ≥30. RNA-Seq reads were aligned to the *Homo sapiens* genome (GRCh38) and downloaded *via* ENSEMBL using Hisat2 (47). After the alignment, the software FeatureCount was used to count reads mapping to genes annotation files (48). For differential expression analysis of RNA-seq results, the edgeR package was used to measure gene expression levels by normalizing the raw counts to counts per million (CPM) and to identify differentially expressed genes between sample groups (49).

### Bioinformatic analysis

Gene set enrichment analysis (GSEA) and pathway enrichment were performed with the STRING online platform (string-db.org) (50). Further analysis was performed in R (v.4.3.2, R Core Team). Enrichments of viral protein and viral RNA interactors in proteomics results were obtained by first matching significant proteins to a manually compiled compilation of viral protein/RNA interactors, then calculating enrichment with Fisher's exact test. Viral protein interactors were compiled based on a series of published large-scale GFP-pulldown experiments (51, 52, 53, 54). Viral RNA interactors were obtained from our recent review article (55). Enrichment analysis of viral protein/RNA interactors in phosphoproteomics results was performed by first obtaining proteins that contain IFN-α regulated phosphosites (FDR <0.05 in moderated T or Z statistics), then matching to the compilation of viral protein/RNA interactors described above and calculated with Fisher's exact test.

Matching of IFN-related GO annotation was performed with package GO.db (https://bioconductor.org/packages/GO.db/) and biomaRt (56). GO terms containing keywords “innate immune/type I interferon/interferon-alpha” were considered IFN-related terms. Genes annotated with these terms were extracted and matched to proteomics results. Matching of ISG interactors was performed using proteins with FDR <0.05 in a large-scale proteomic-based interactor screening study (57). Matching of FACS-screened ISGs was performed using proteins with Z < −1.5 from screening results (58, 59).

Kinase-substrate enrichment analysis (KSEA) was performed following the procedure described in published studies (60, 61). Briefly, kinase-substrate correlation annotations were manually compiled from a series of online databases (PhosphoSitePlus (62), PhosphoELM (63), SIGNOR (64), DEPOD (65), iPTMnet (66), NetworKIN and Netphorest (67)). After matching to phosphoproteome quantification and semi-quantification results, the activity of each kinase was calculated by the average fold change of phosphosites that it can regulate. Kinases of significantly altered activities were determined by Z-statistics of all kinase activity values, with a cutoff set at FDR <0.05.

Distances between phosphosites and RNA-binding domains (RBDs) were calculated based on RNA-interactive peptide fragments (RBDpep) in RBDmap (68). Briefly, RBDpep peptide fragments in each RBP were assembled into non-overlapping RNA binding regions, the distance of a site was set to zero if it was located inside RNA binding regions, and determined as the number of amino acids between the site and the closest RNA binding region. Distances of random distributed sites were calculated as follows: for sites located inside the RNA binding region, distances remained zero; for sites located between two RNA binding regions, distances were calculated as expected values when site positions distributed randomly within those sequences.

## Results

### Widespread Proteome Differences Between HEK293 and HEK293T

**Fig. 1:**
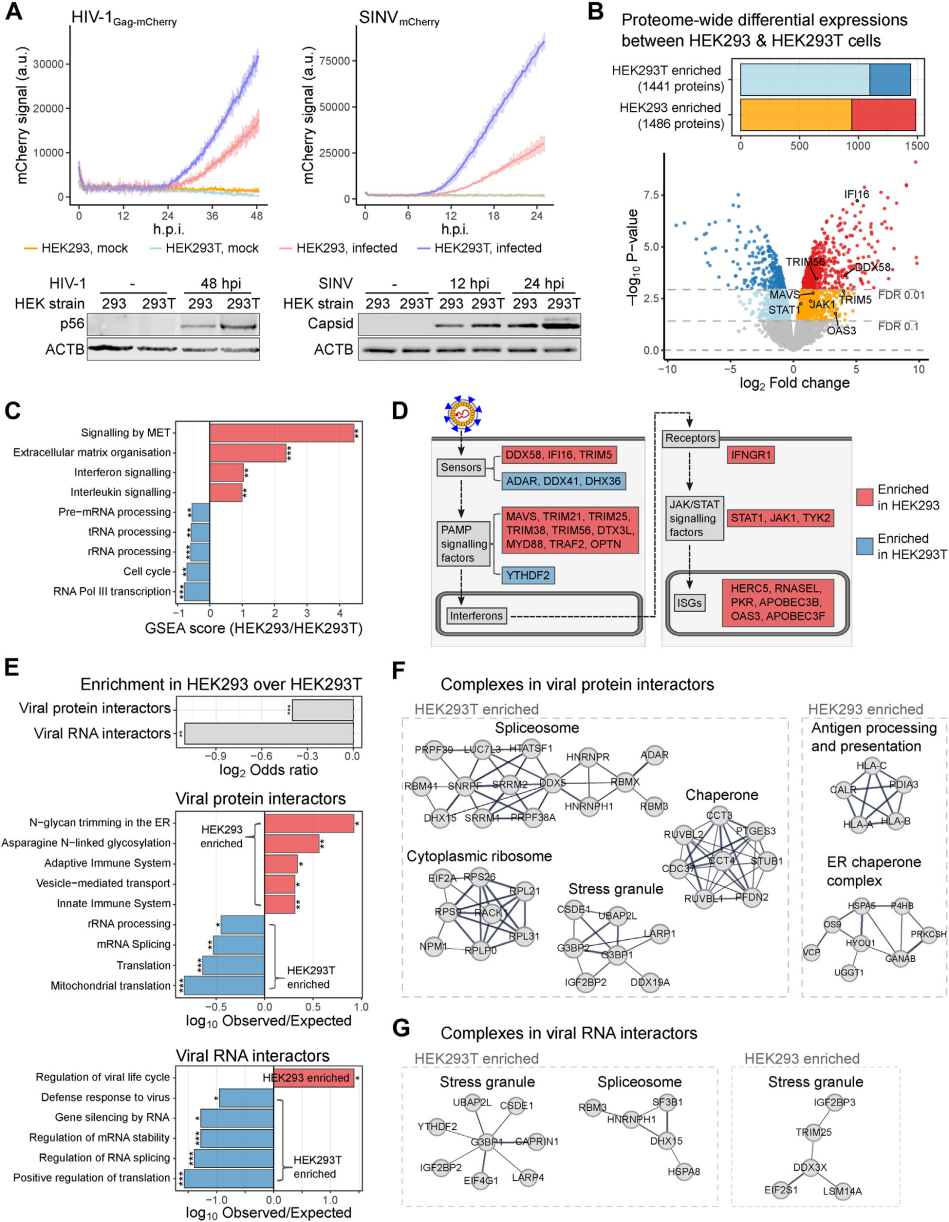
**Host factors and processes underlying different permissiveness between HEK293 and HEK293T cells**. *A*, infection fitness of HIV-1_Gag-mCherry_ (*left*) and SINV_mCherry_ (*right*) in HEK293 and HEK293T cells. Fluorescence signals were measured every 15 min in culture conditions using a plate reader (*top*, n = 3, multiplicity of infection (MOI) = 1). Western blottings were performed using HIV-1_Nef-mCherry_ (*left*) and SINV_mCherry_ (*right*) with antibodies of viral capsid proteins (*bottom*, MOI = 1). *B*, the result of quantitative proteomic comparison between HEK293 and HEK293T. Bar plot summarised numbers of proteins with FDR <0.1 and FDR <0.01 in each cell line (*top*). Volcano plot showed log_2_ fold change and significance (*p*-value) of each protein between HEK293 and HEK293T cells (*bottom*, supplemental Table S1). *C*, differentially enriched *Reactome* pathways in Gene set enrichment analysis (GSEA). *p*-values of enrichments were ∗ labelled (∗∗*p* < 0.01, ∗∗∗*p* < 0.001). *D*, a schematic of the innate immune response against the virus with key processes labeled in *grey boxes*. Differentially enriched genes in each process were labeled red (HEK293-enriched) or *blue* (HEK293T-enriched). *E*, enrichments of viral protein interactors and viral RNA interactors between HEK293 and HEK293T cells (*top*), and Gene Ontology–Biological processes (GO-BP) terms enrichments of viral protein interactors (*mid*) viral RNA interactors (*bottom*) in HEK293 (*red*) and HEK293T (*blue*) cells. *p*-value represented by ∗ label (∗*p* < 0.05, ∗∗*p* < 0.01, ∗∗∗*p* < 0.001). *F*, protein-protein interaction complexes among viral protein interactors differentially enriched between HEK293 and HEK293T cells. *G*, as in *F* but for viral RNA interactors.

Previous work reported that infection of multiple viruses, including HIV-1, proceeds more efficiently in HEK293T cells than in HEK293 cells. To further investigate these results, we infected HEK293 and HEK293T with an HIV-1_Gag-mCherry_ replicon (supplemental Fig. S1*A*) pseudotyped with the glycoprotein of vesicular stomatitis virus (VSV-G), and viral gene expression was measured using mCherry as a proxy as in (42). We observed a substantially higher HIV-1_Gag-mCherry_-derived fluorescent signal in HEK293T than in HEK293 cells throughout the course of infection (Fig. 1*A*, top). Consistently, abundance of Gag and p24 was higher in HEK293T than in HEK293 cells for both HIV-1_Gag-mCherry_ or HIV-1_Nef-mCherry_ (Fig 1*A*, bottom; supplemental Fig. S1*B*) (69). To generalize these results, we infected HEK293 and HEK293T with SINV, a vector-borne RNA virus within the Togaviridae family (SINV_mCherry_; supplemental Fig. S1*A*) (42). Analysis of mCherry and capsid abundance confirmed that SINV infects more efficiently HEK293T than HEK293 (Fig 1*A* and supplemental Fig. S1*B*). Altogether, these results and those reported previously (25, 26, 27, 28, 29, 30, 31, 32, 33) support that HEK293T are more permissive to virus infection than HEK293.

To explore factors for the differential ability of HEK293 and HEK293T cells to sustain infection, we conducted whole-cell proteome profiling with stable isotope labeling with amino acid in culture (SILAC) and offline high-pH RP fractionation, achieving over 99% isotope incorporation (supplemental Fig. S1*C*) (70, 71). We identified and quantified 8553 and 7558 proteins, respectively (supplemental Fig. S1, *D* and *E*). Despite the shared genetic background of the two lines, ∼3k proteins exhibited differential abundance at 10% false discovery rate (FDR), with 1486 and 1441 proteins being enriched in HEK293 and HEK293T cells, respectively (Fig. 1*B*). Gene set enrichment analysis (GSEA) revealed a prevalence of interferon signaling pathway in HEK293 cells over HEK293T cells (Fig. 1*C*). Further investigation identified proteins involved in virtually all processes of the antiviral response that are more abundant in HEK293 cells than in HEK239T (Fig. 1*D*). These included: (i) intracellular sensors such as RIG-I (DDX58) and IFI16 (42, 72, 73), (ii) effectors such as TRIM5, PKR, OAS3, and RNASEL (74, 75, 76), (iii) signal transducers such as MAVS and TRAF2 (77), iii) receptor signaling factors such as MYD88 and the tyrosine kinases JAK1 and TYK2 (78, 79), and (iv) the transcription factor STAT1 (80).

The widespread depletion of innate immunity proteins in HEK293T cells implies a globally dysfunctional antiviral response that cannot be ascribed to individual proteins (25). To verify this, we treated both cell lines with IFN-α and measured the level of STAT1 Y701 phosphorylation (supplemental Fig. S1*F*). Interestingly, blotting results showed that both cell lines respond to IFN-α stimulation, but with a different strength (supplemental Fig. S1*G*), and saturation dose (supplemental Fig. S1*H*). Differences in strength and saturation point of the IFN-α stimulation are presumably derived from the distinct stoichiometry of the IFN receptors and signaling proteins in the two lines.

Beyond innate immunity, hundreds of proteins involved in other cellular processes were also differentially expressed in HEK293 and HEK293T cells. To identify regulators of infection that are outside the scope of well-characterized ISGs, we cross-referenced our dataset with a manually compiled and curated list of viral protein and RNA interactors from previous publications (supplemental Fig. S1*I*) (51, 52, 53, 54, 55). Viral protein and RNA interactors in HEK293T are enriched in gene ontology (GO) terms related to RNA metabolic processes that are typically classified as dependency factors, including the translation and splicing apparatus and RNA stability factors (Fig. 1, *E* and *F*). Conversely, the viral protein interactors in HEK293 cells are involved in immune response and protein N-glycosylation (Fig. 1*E*, mid), including MHC-I and MHC-I peptide loading complex (Fig. 1*F*, right). Notably, non-core stress granule components showed different abundance in HEK293 and HEK293T (Fig. 1*G*). YTHDF2 is enriched in HEK293T and was reported to inhibit innate immune response through RNA methylation-dependent degradation (81). By contrast, TRIM25 and LSM14A are associated with antiviral functions and are enriched in HEK293 (82, 83). Altogether, our data support a model in which the distinct permissiveness of HEK293 and HEK293T is derived from complex proteome differences involving a wide range of antiviral and dependency factors.

### The Proteome Landscape of HEK293 cells after IFN-α Stimulation

**Fig. 2:**
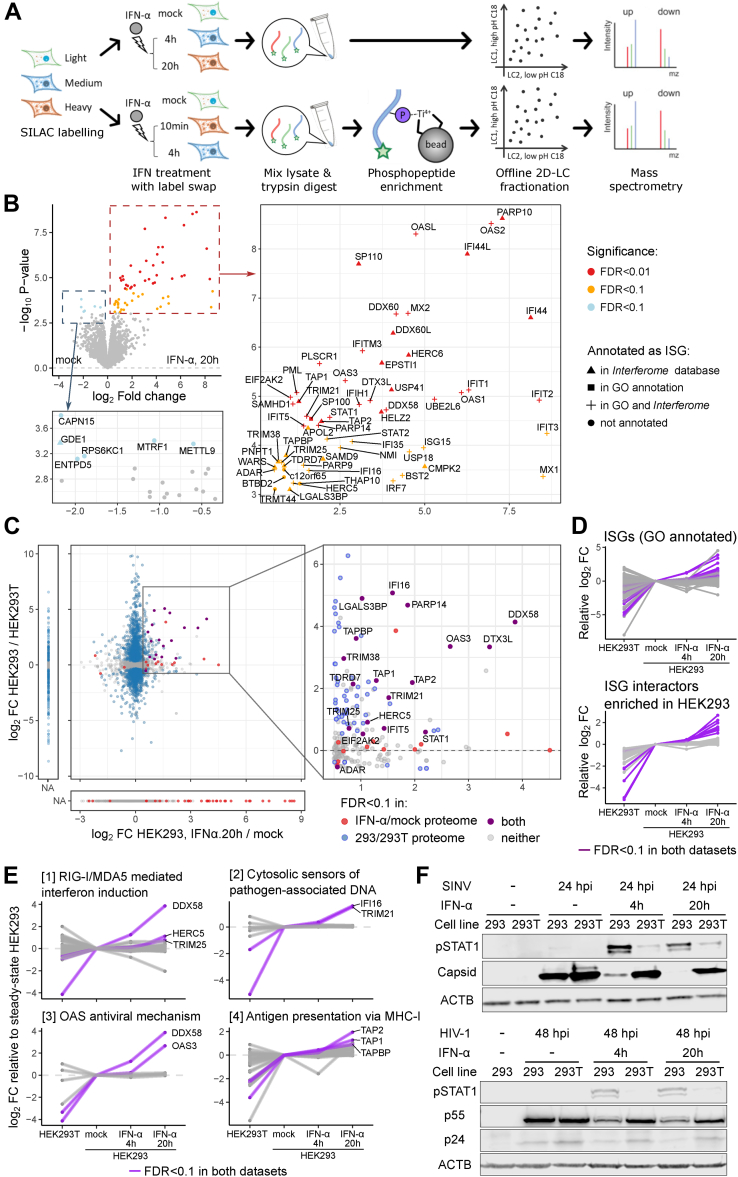
**Proteome landscape of HEK293 cells after IFN-α stimulation**. *A*, schematic workflow of proteome and phosphoproteome profiling of IFN-α stimulated HEK293 cells. SILAC samples were mixed with label swap between replicates. *B*, Volcano plot with text label showing proteins with FDR <0.1 after 20 h IFN-α treatment (supplemental Table S2). Proteins related to IFN-α were drawn in different shapes depending on the source of annotations – triangle (Δ): annotated in microarray (MA) studies in database *Interferome*; square (□): annotated in GO-BP terms; plus (+): annotated in both MA studies and GO-BP terms; circle (○): no IFN-α related annotation in either source. *C*, scatter plot comparing log_2_ fold changes quantified in IFN-α/mock proteome and HEK293/HEK293T proteome for each protein. *D*, parallel coordinate plots showing protein expression patterns across cellular states. Proteins with FDR <0.1 in both IFN-α/mock and HEK293/HEK293T proteomes were color-labeled. *E*, as in *D* but showing proteins in pathways of interest. *F*, Western blotting with antibodies of STAT1-pTyr701 (pSTAT1) and viral capsid proteins. Cells were treated with 200 U/ml IFN-α for indicated time period, then infected at an MOI = 1 with SINVmCherry (*top*) or HIV-1Nef-mCherry (*bottom*).

We next extended the comparative proteomics analysis to include a virus-hostile cellular state. We focused on IFN-α treated HEK293 given that HEK293T cells possess a compromised antiviral activity even in the presence of IFN-α (Fig 2*F* and supplemental Fig. S2*A*). We observed a mild but significant inhibition of SINV gene expression just with 10 min of IFN-α treatment prior to infection, which increased in magnitude when the pre-treatment was extended to 4 and 20 h (supplemental Fig. S2, *B* and *C*). To profile the proteome responses, SILAC-labelled HEK293 cells were treated with mock or 200 U/ml IFN-α for 10 min, 4 h, or 20 h (Fig. 2*A*). Proteome changes are expected to require hours, while post-translational modifications (PTMs) can occur within minutes. Therefore, we used the 10 min and 4 h samples for phosphoproteomics and the 4 and 20 h samples for deep proteome analyses (Fig. 2*A*).

Deep proteome profiling identified and quantified 10,154 and 9131 proteins (supplemental Fig. S2, *D*–*F*). To our surprise, only 66 proteins showed significant changes at 20 hpt (hours post-treatment) under a cut-off of FDR <0.1 (Fig. 2*B*). This is a small number given that 7350 human genes are reported to respond to type I IFN in microarray (MA)-based studies in the *Interferome* database (84). Among the proteins with significant upregulation upon IFN-α treatment, 37 were classified as ISGs by GO annotation, 55 by MA, and 36 by both (Fig. 2*B*). Four proteins were not linked to IFN-α response according to the referenced sources. By contrast, none of the IFN-α downregulated proteins were related to innate immunity except for GDE1 which was detected in 1 MA study (85). The depth and precision of this proteome dataset also allowed the depiction of temporal changes across early (4 hpt), and late (20 hpt) time points. Quantified proteins were divided into 8 groups based on their temporal profile (supplemental Fig. S2*G*). Groups showing quick and continuous or late induction upon IFN-α stimulation were enriched in antiviral response pathways, including RIG-I/MDA signaling, IFN-α signaling, and ISG15-mediated antiviral mechanisms (supplemental Fig. S2*H*). Several known antiviral proteins were present in a third group showing quick induction followed by plateau, including the transcription factor IRF9 and RIG-I (DHX58) (supplemental Fig. S2*G*). Most proteins in the other groups have no known role in innate immunity.

To search for factors that modulate virus permissiveness across different cellular states, we compared proteomic data from permissive HEK293T cells, steady-state HEK293 cells, and hostile IFN-α stimulated HEK293 cells (Fig. 2*C*). We found 17 proteins enriched in virus-restrictive IFN-α stimulated HEK293 cells and depleted in virus-permissive HEK293T (Fig. 2*C*). Thirteen of these are functionally well-annotated ISGs, suggesting that these may represent a pivotal antiviral network defining cell susceptibility to virus infection. These ISGs displayed a gradual increase in protein abundance as the cellular state becomes more virus-restrictive (Fig. 2*D*, top). Pathway analysis determined that they are involved in viral RNA and DNA sensing, and 2′-5′-oligoadenylate synthesis (Fig. 2*E*). Moreover, we observed several proteins described as interactors of ISGs that also increased in abundance as cells become more restrictive (Fig. 2*D*, bottom) (57). These ISG interactors include LGALS3BP which is a scaffold protein for PAMP signaling with broad-spectrum antiviral roles (86), and TAP1/2 are antigen presentation factors that were linked to innate immunity in functional studies (87, 88); however, their roles in IFN response are not well understood.

### Transcriptome and Proteome Profiling Reveals Discordances Between RNA and Protein Levels During IFN-α Response

**Fig. 3:**
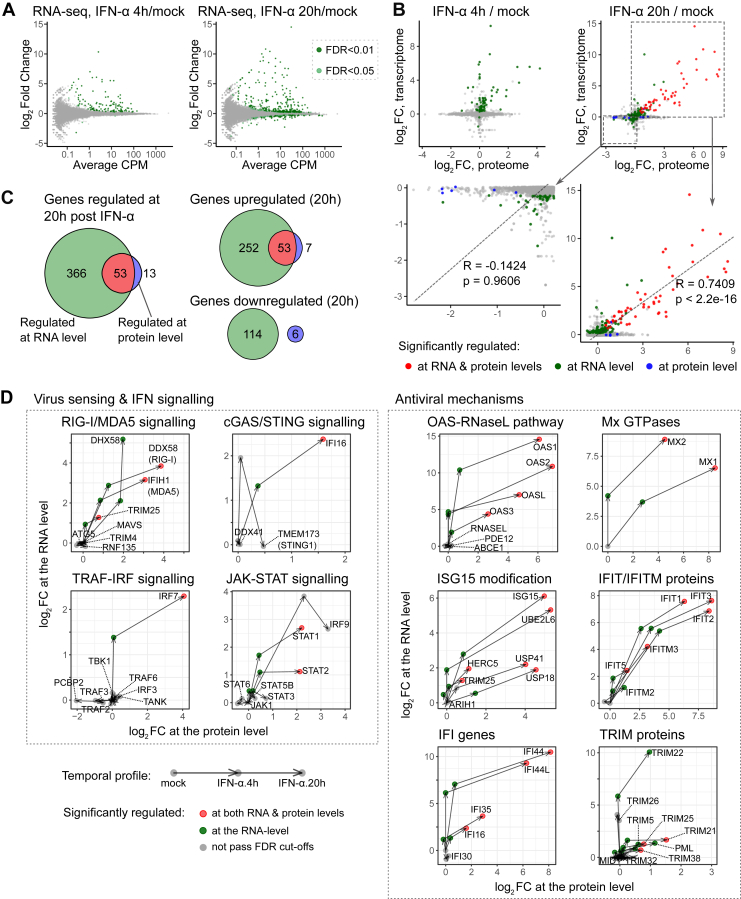
**Transcriptome analysis of HEK293 cells after IFN-α stimulation**. *A*, MA plots of IFN-α induced transcriptome changes at 4 hpt (*left*) and 20 hpt (*right*) in HEK293 cells. Gene expression was measured by CPM (counts per million reads), referring to the number of reads mapped to transcript scaled by the number of sequencing reads (supplemental Table S3). *B*, scatter plots comparing log_2_ fold changes in transcriptome and proteome after IFN-α stimulation. Only genes quantified in both datasets were plotted. *C*, Venn diagrams comparing numbers of IFN-α induced significant changes at 20 hpt at RNA and protein levels. *D*, Vector plots visualizing temporal expression profiles after IFN-α stimulation at RNA and protein levels.

To investigate the relationship between transcript and protein presence, we performed RNA-seq with IFN-α stimulated and untreated HEK293 cells. Our results revealed that 126 and 419 genes exhibited significant differential expression at 4 and 20 hpt, respectively (Fig 3*A* and supplemental Fig. S3*A*). Comparing this RNA-seq result with similar studies in different human cell lines, we found that numbers of significantly up- and downregulated genes vary substantially across datasets (supplemental Fig. S3*B*), which can reflect differences in cell types and experimental conditions (89, 90). Despite these quantitative differences, we observed high correlations among genes that were commonly upregulated across cell types (R = 0.63 and 0.73, supplemental Fig. S3*C*). In addition, a large proportion of commonly upregulated genes are GO-annotated ISGs (41%), while less than 4% of genes that exhibited cell type-specific regulations are linked to IFN response (supplemental Fig. S3*D*). These observations support that IFN-α responses in HEK293 cells are representative of ISGs with robust upregulation across cell types.

We next compared our transcriptome and proteome datasets to assess correlations between RNA and protein levels at different time points. When focusing exclusively on genes upregulated at the RNA and protein level, we observed a positive trend at both 4 and 20 hpt (R = 0.74, Fig. 3*B*, bottom-right). When equal weighting was applied to all genes and also considered genes only quantified by one method, we found that 366 out of the 419 genes regulated at the RNA level had no corresponding changes at the protein level (Fig. 3*C*, left). We observed a good correlation between RNA and protein for 53 upregulated genes, mostly annotated ISGs. Surprisingly, we noticed a striking lack of correlation between RNA and protein for downregulated genes (Fig. 3, *B* and *C*). This discordance was also observed when using other published RNA-seq experiments (supplemental Fig. S3*B*) (89, 90). We next assessed if the observed RNA-protein discordance was caused by different detection limits between RNA-seq and proteomics. Interestingly, ∼40% of the genes with changes only at the RNA level were also quantified by our proteomic analysis (supplemental Fig. S3*E*). This indicates that a substantial proportion of RNA-level changes are not reflected at the protein level, among which we observed a series of well-studied ISGs such as IRF1/2, IFI6/27 (supplemental Fig. S3*F*) (91, 92, 93).

To better understand the gene expression kinetics during IFN-α response, we visualized temporal kinetics profiles with vector plots connecting RNA- and protein-level changes at different time points on the same coordinate map (Fig. 3*D*). Most genes within the strongest upregulated pathways displayed similar temporal kinetics. These genes first increase at the RNA level, followed by an upregulation of both RNA and protein at 20 hpt (Fig. 3*D*). These profiles agree with a transcription/translation-driven gene expression model, that requires time to transform the transcriptional response into protein (94). This temporal gap should be considered when analyzing IFN responses with RNA-centric methods.

### Protein-Level IFN-α Response has a Higher Correlation With Characterized Antiviral Functions

**Fig. 4:**
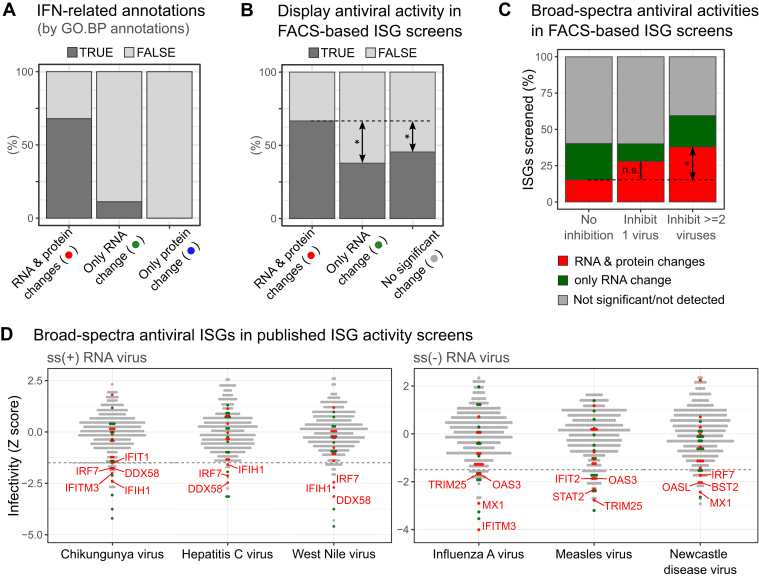
**Correlation analysis with characterized antiviral functions**. *A*, Proportions of GO-BP annotated ISGs among genes regulated by IFN-α in HEK293 cells at RNA/protein levels. *B*, proportions of antiviral ISGs verified by FACS screening studies (summarised in Table 1) among genes regulated by IFN-α in HEK293 cells at RNA/protein levels. *C*, proportions of broad-spectrum antiviral ISGs among genes regulated by IFN-α in HEK293 cells. ISG activities in FACS screening results were grouped by the number of viruses they can inhibit: no inhibition, inhibit 1 virus (virus-specific), and inhibit ≥2 viruses (broad-spectra). *D*, dot plots of FACS screening results that are summarised in Table 1. Genes with broad-spectrum antiviral activities were color-labeled based on IFN-α response at RNA/protein levels in HEK293 cells. ISGs with Z < −1.5 for each virus were text labeled.

We then assessed functional annotations for genes that display discordance between RNA and protein levels. Indeed, we noticed that genes regulated at both RNA and protein levels were heavily annotated with IFN-related GO terms, while the opposite was observed with genes exhibiting changes only at RNA or protein level (Fig. 4*A*). One major limitation of annotation-based analysis is its high dependency on the quality of annotation. We thus complemented the classic ontology analysis with experimental data including large-scale screenings that systematically assessed the antiviral activity of nearly 400 ISGs against 17 RNA viruses (Table 1) (58, 59). We found a prevalence of ISG with experimentally determined antiviral activity within the group of proteins upregulated at both RNA and protein levels (Fig. 4*B*). These ISG screenings also highlighted a group of broad-acting ISGs with inhibitory effects for multiple viruses spanning several families (59). These ISGs are pivotal in the antiviral response and include well-characterized sensors (RIG-I/DDX58, MDA5/IFIH1), transcription factors (IRF and STAT families), and effectors (OAS, MX, and IFI families). Strikingly, these broad-spectrum ISGs are more prevalent within the genes with correlation at the protein and RNA levels than in the discordant genes (Fig 4, *C* and *D* and supplemental Fig. S4*B*). Another large-scale study defined the “core ISG set”, which included genes with robust stimulation in response to type-I IFN across mammals (89). Again, the genes with robust upregulation at the RNA and protein levels are enriched in “core-ISGs” when compared with the discordant genes (supplemental Fig. S4*A*). Altogether, our results pinpoint a strong correlation between pivotal antiviral factors and the robust response to IFN stimulation involving both RNA and protein.

**Table 1:** ISGs assessed in fluorescence-activated cell sorting (FACS) based antiviral activity screenings

Virus type	Virus	ISGs screened	Publication
ss(+) RNA virus	HCV	360	Schoggins, et.al., 2011 (59)
	YFV	360	
	WNV	372	
	VEEV	372	
	CHIKV	367	
	PV	372	Schoggins, et.al., 2014 (58)
	CVB	367	
	EAV	351	
	SINV	341	
	ONNV	337	
ss(−) RNA virus	IAV	370	
	PIV3	363	
	NDV	364	
	HMPV	360	
	RSV	358	
	MV	356	
	BUNV	331	

We next focused on the differentially regulated genes with no IFN-related GO annotation. We noticed that many of these poorly understood genes clustered together in a protein-protein interaction network, suggesting functional interconnections (supplemental Fig. S4*C*). Several highly connected hubs (nodes with ≥5 connections) in this network included CMPK2 and IFI44, which have recently been reported as antiviral factors (95, 96). HERC6 is another network hub, which is a catalytically inactive homolog of the known antiviral factor HERC5 (97) with no yet described function in immunity. Interestingly, genes regulated only at the RNA level were enriched in apoptosis factors (supplemental Fig. S4*D*). Proteome data confirmed that many key regulators of apoptosis were quantified but had no significant changes at the protein level upon IFN-α stimulation, as it is the case of the pivotal apoptotic proteases CASP4, 7, and 8 (98) (supplemental Fig. S4*E*). These genes showed a distinct induction profile when compared with ISGs, with low to no increase at 4 hpt and a strong upregulation at 20 hpt (supplemental Fig. S4*F*). A plausible explanation for these results is that ISGs represent a first transcriptional wave, followed by a second wave including the apoptotic program if the IFN-α stimulation is prologued in time. The delayed production of mRNAs encoding pro-apoptotic factors would inevitably result in the accumulation of these proteins at later times post-exposure, which were not covered in our proteomic analysis.

### Phosphoproteomics Links IFN-α Response to RNA-Binding Proteins

**Fig. 5:**
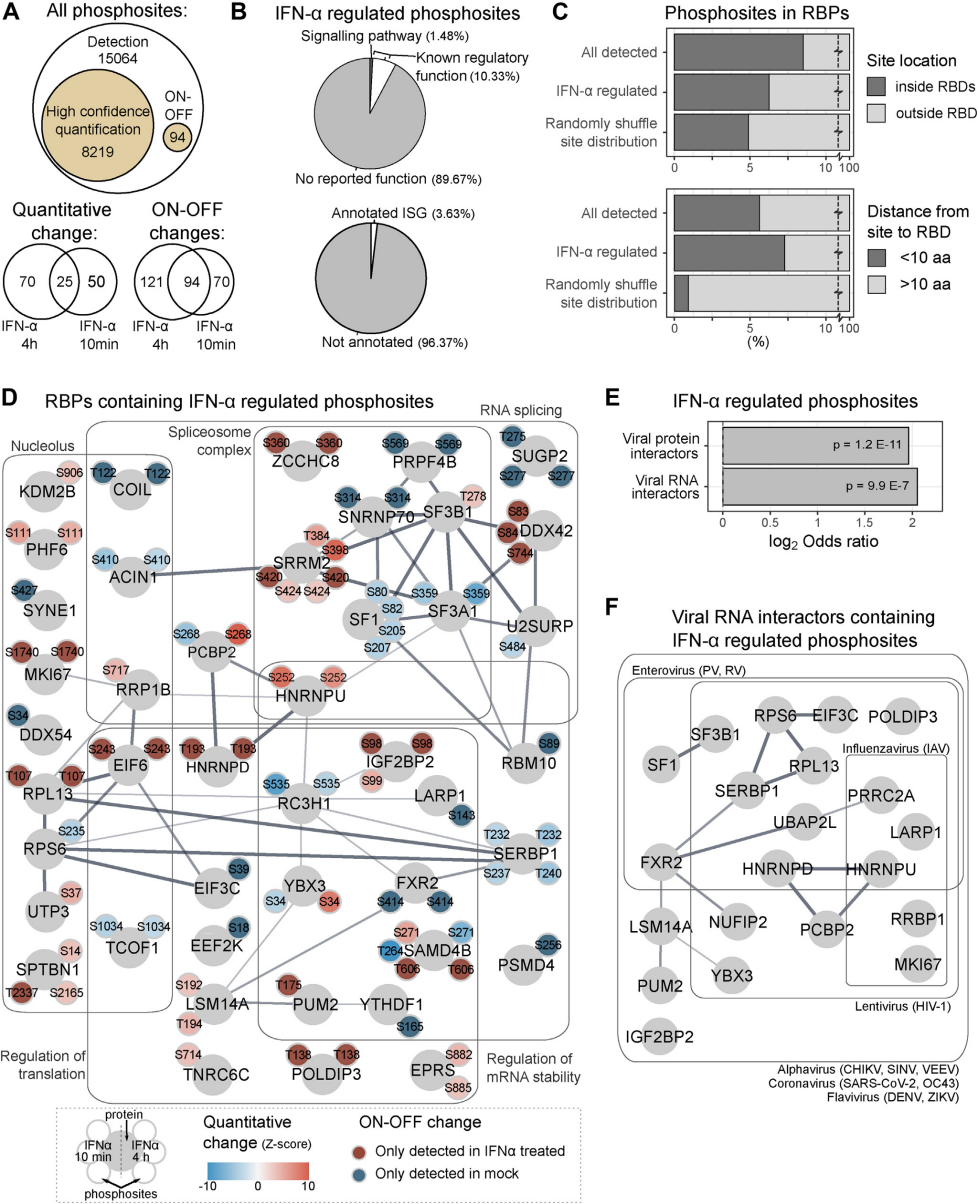
**Phosphoproteome analysis of HEK293 cells after IFN-α stimulation**. *A*, Venn diagrams summarising numbers of phosphosites identified, quantified, and IFN-α regulated in phosphoproteomic result that combined data from two instrument configurations (supplemental Table S4). *B*, proportions of functional annotations among IFN-α regulated sites (*top*) and IFN-related GO-BP annotations among phosphoproteins that contain IFN-α regulated phosphosites (*bottom*). *C*, proportions of phosphosites that are located inside annotated RNA-binding domains (RBDs; *top*), or located within 10 amino acids before or after RBDs (*bottom*). *D*, Protein-protein interaction network of RNA-binding proteins (RBPs) that contain IFN-α regulated phosphosites. Positions of phosphosites were labeled with small circles adjacent to each RBP. *E*, enrichments of viral protein interactors and viral RNA interactors among proteins that have IFN-α regulated phosphosites. *F*, protein-protein interaction network of viral RNA interactors that have IFN-α regulated phosphosites. The virus family and species that each protein interacts with were text-labeled.

To assess if post-translational modulations contribute to defining cell permissiveness, a deep phosphoproteome analysis was conducted in cells treated with IFN-α for 10 min or 4 h (Fig. 2*A*). We identified 23,377 phosphosites in 3942 proteins (supplemental Table S4 and supplemental Fig. S5, *A*–*E*). Interestingly, most of the phosphosite alterations had no matching changes in protein abundance (supplemental Fig. S5*F*), which indicates that phosphorylation may represent an additional regulatory layer in IFN-α treated cells that is independent of protein abundance. Our analysis identified 145 phosphosites with altered abundance, and an additional 285 sites with “ON-OFF” changes (Fig 5*A* and supplemental Fig. S5*E*). When mapped to functional annotation databases (62), we found that only 10.3% of IFN-α regulated sites have supporting regulatory functions, 1.5% have known roles in signaling pathways, and 3.6% map to known ISGs (Fig. 5*B*). A similar proportion of functional annotation was also observed for the total set of identified phosphopeptides, irrespective of IFN-α regulation (supplemental Fig. S5*H*). These results are consistent with a published meta-analysis suggesting that the vast majority of recorded phosphosites have no reported function (99). Strikingly, we detected changes in key nodes of the innate immune signaling, including the nuclear body-associated protein PML (100) and ANKRD17 (101) (supplemental Fig. S5*H*). We further investigated processes underlying the observed phosphosite regulations using kinase-substrate enrichment analysis (KSEA) (60). We mapped 11,305 kinase-substrate connections across 390 kinases and 4698 sites, uncovering 18 kinases with significantly altered activities (supplemental Fig. S5*I* and supplemental Table S5). A few of these kinases are linked to immune response, including IKBKE which activates IRF3 and STAT proteins (102), and WNK kinases (51).

RNA-binding proteins (RBPs) are central to virus infection acting as dependency and antiviral factors (103). Critically, their RNA-binding domains (RBDs) are enriched in PTMs, which can modulate the interaction with RNA (68). Here we assessed if phosphorylation could regulate cellular RBPs during IFN-α response. Phosphosites detected in our dataset were indeed prevalent inside known RBDs, although this enrichment was more moderate when considering only the IFN-α regulated phosphosites (Fig. 5*C*, top). Strikingly, IFN-α modulated phosphosites became strongly enriched when RBDs were considering the 10 amino acids before and after the RBD (Fig 5*C*, bottom and supplemental Fig. S5*J*). The proximity between IFN-α regulated sites and RBDs indicates that phosphorylation-dependent regulation of RBP could play a role in IFN-α response. Protein-protein interaction network revealed that RBPs containing IFN-α regulated sites were widely involved in gene expression control, including regulation of splicing, translation, and RNA degradation (Fig. 5*D*). Cross-referencing analysis confirmed significant enrichments of IFN-α regulated phosphoproteins among known interactors of viral RNA and proteins, with several of them being capable to bind the RNA of viruses from different species and families (Fig. 5, *E* and *F*). One of these broad-spectrum interactors, HNRNPD, was phosphorylated after IFN-α treatment at T193, which localizes to its RBD (supplemental Fig. S5*K*); while POLDIP3 was phosphorylated at T138 close to its non-canonical RBD (supplemental Fig. S5*K*). These observations implied that interactions with viral RNA could be subject to phosphorylation-dependent regulation during IFN-α response.

### Uncovering New Regulators of Virus Infection

**Fig. 6:**
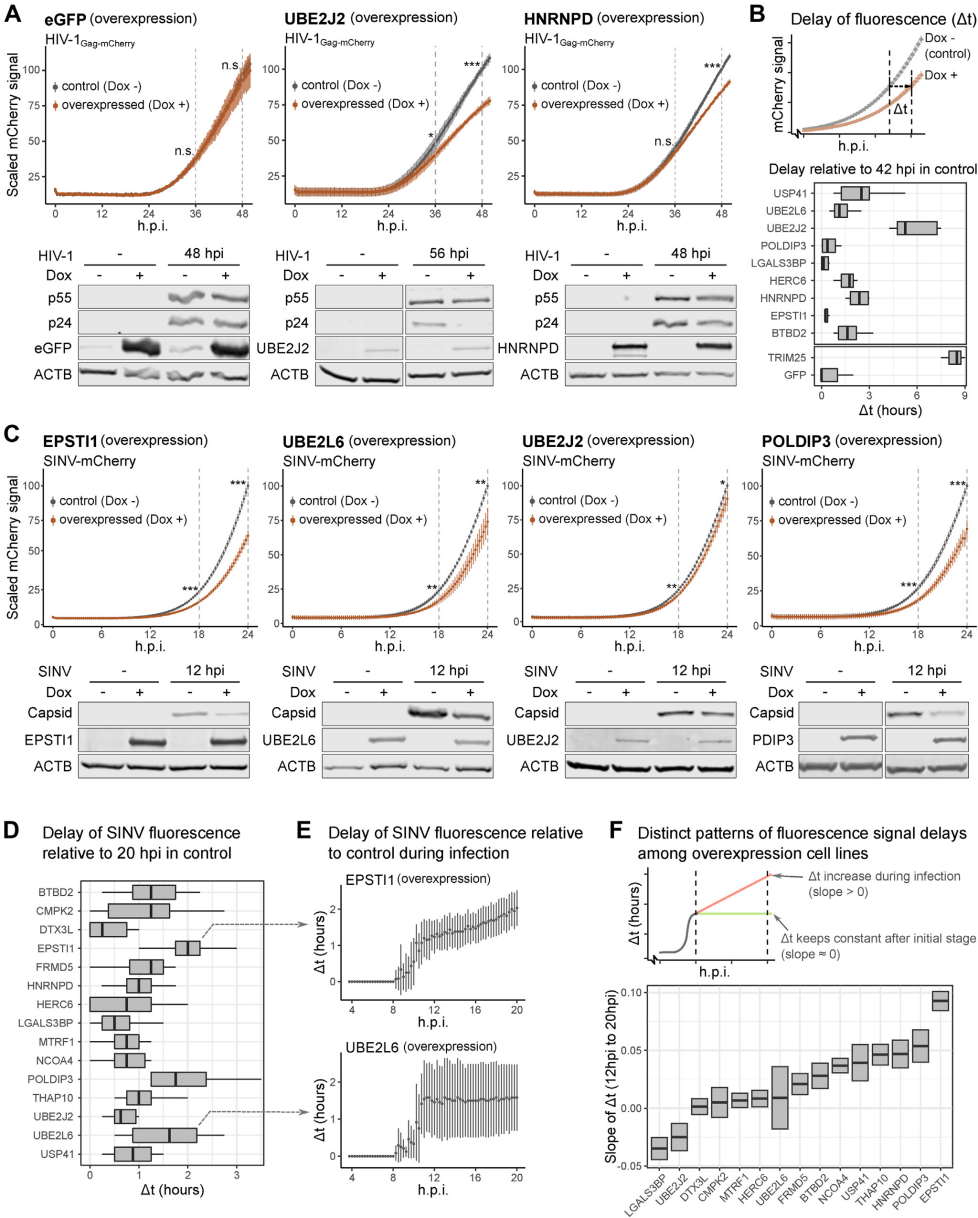
**Virus-inhibitory effects against SINV and HIV-1 in stable cell lines expressing candidate genes**. *A*, infection fitness of HIV-1_Gag-mCherry_ in HEK293 inducible stable lines expressing candidate genes with eGFP fusion (*top*, MOI = 1, n = 3). Fluorescence signals were measured as in Fig 1*A*. Western blotting analyses were performed with anti-eGFP and anti-p24 (bottom, MOI = 1). *B*, schematic of fluorescence signal delay analysis that assesses delays in virus gene expression caused by expression of candidate gene (*top*), and delay analysis results at 42 hpi for all candidates assessed with HIV-1_Gag-mCherry_ (*bottom*). *C*, as in *A* but with SINV_mCherry_ for fluorescence assay and Western blots. *D*, as in *B* but with SINV_mCherry_ at 20 hpi. *E*, Fluorescence signal delay analysis from 4 to 20 hpi for EPSTI1 and UBE2L6 overexpression cells. *F*, schematic of delay pattern analysis that assesses slope of delays (*top*), and comparison of delay patterns between 8 and 20 hpi for all candidates assessed with SINV_mCherry_ (*bottom*).

To test if additional antiviral proteins exist beyond known ISGs, we selected 15 candidates with differential abundance or phosphorylation status in IFN-α, prioritizing proteins with little or no previous implications in immunity (supplemental Table S6). These candidates were expressed in a doxycycline-dependent manner fused to eGFP and used to challenge HIV-1_Gag-mCherry_ and SINV_mcherry_ infection (supplemental Fig. S6*A*). We used TRIM25-eGFP and eGFP as positive and negative controls, respectively (42). A significant decrease of red fluorescence signal derived from HIV-1_Gag-mCherry_ was observed for 6 out of 9 tested genes (Fig 6*A* and supplemental Fig. S6, *B* and *C*). Five of these phenotypes were confirmed by analysis of Gag/p24 expression by Western blotting (Fig 6*A* and supplemental Fig. S6*D*). Interestingly, both E2 ubiquitin ligases UBE2L6 and UBE2J2 showed an inhibitory effect against HIV-1 (Fig. 6*A* and supplemental Fig. S6*D*). While UBE2L6 is a known ISG15 ligase in innate immune response (104), UBE2J2 has no documented role in HIV-1 infection. However, UBE2J2 reduced to nearly half the expression of p24 (Fig. 6*A* and supplemental Fig. S6*D*). Nuclear-localized RBP HNRNPD harbor an IFN-α induced phosphosite and, interestingly, our data showed that its overexpression can suppress HIV-1 replication (Fig. 6*A*).

To quantitatively evaluate HIV-1 inhibitory effects, we assessed the delay in viral gene expression caused by the overexpression of the candidates in our near real-time plate reader assay (Fig. 6*B*, top). We first determined the mCherry fluorescence signal at 42 hpi in the control cells and then assessed the time such signal levels were achieved in the different overexpression lines, which we refer to as “delay” or Δt. Untagged eGFP expression caused a delay relative to the uninduced line that was negligible, similar to EPSTI1, LGALS3BP, and POLDIP3 expression. However, the expression of several eGFP-fused candidates led to delays ranging from 2 to 5 h, with UBE2J2 causing a delay comparable to TRIM25 (Fig. 6*B*, bottom). We then extended the delay analysis to the 12 ∼ 42 hpi window. GFP control and non-inhibitory candidates showed near a 0 h delay throughout this time window, while fusion proteins with anti-HIV-1 effects had continuous increases in Δt after 24 hpi (supplemental Fig. S6*E*).

We next extended the study to a virus from a different family, SINV_mCherry_. Interestingly, 13 out of 15 tested fusion proteins caused a statistically significant decrease in mCherry fluorescent signal compared to the uninduced cells (Fig 6*C* and supplemental Fig. S6, *F* and *G*). The inhibitory phenotypes of 5 fusion proteins were confirmed by Western blots (Fig. 6*C* and supplemental Fig. S6*H*). EPSTI1, which showed no effect on HIV-1, caused the strongest anti-SINV phenotype (Fig. 6*C*). Interestingly, the antiviral activity of EPSTI1 was reported for other positive sense, single-stranded RNA virus, hepatitis C virus (105). Consistently with the HIV-1 data, overexpression of the E2 ligases UBE2J2 and UBE2L6 promoted a significant inhibition of SINV_mCherry_ (Fig. 6*C*). Overexpression of HNRNPD and POLDIP3, both containing IFN-α regulated phosphosites, also inhibited SINV gene expression (Fig. 6*C* and supplemental Fig. S6*F*).

Most proteins caused a delay in SINV_mCherry_ that ranged from 1 to 2 h, with EPSTI1, POLDIP3, and UBE2L6 promoting the strongest effects (Fig. 6*D*). When extended to the 4 ∼ 20 hpi window, we noticed distinct delay patterns across candidates. EPSTI1 overexpression led to a continuous increase in Δt, whereas the delay with UBE2L6 overexpression remained constant (Fig. 6*E*). We performed linear regression for Δt for the 12 to 20 hpi window and compared the different delay patterns based on the resulting slope (Fig. 6*F*, top). Six candidates had clear positive slopes (NCOA4, USP41, THAP10, HNRNPD, POLDIP3, EPSTI1), suggesting that viral suppression increases over time (Fig 6*F*, bottom and supplemental Fig. S6*I*). Other proteins showed no substantial increase in Δt after initial inhibition. Altogether, these results imply two different behaviors: while some candidates exert their functions throughout the infection, others limit their action to the early stages of infection allowing viral gene expression recovery.

## Discussion

In this study, we have analyzed the proteome associated with different cellular states, including the permissive HEK293T, intermediate HEK293, and the hostile environment generated upon IFN-α stimulation. Our results highlight the widespread differences between HEK293T and HEK293 cells in their proteome, despite both lines having a shared lineage. Many laboratories have employed HEK293T cells due to their capacity to sustain high virus production, in analogy to other permissive cells such as Vero and BHK-21. Due to their high transfection efficiency and recombinant protein expression capacity, HEK293T cells have also been broadly employed to answer fundamental questions about the molecular biology of viruses (57, 58, 106, 107). Our results demonstrate that in contrast to HEK293, the proteome of HEK293T lacks important cellular proteins that are central players in host-virus interactions, including a wide range of ISGs.

Conversely, the proteome changes in HEK293 associated with the establishment of the antiviral state by IFN-α are limited and focused exclusively on a group of core ISGs that are robustly expressed at the protein level. Most IFN-α studies have used microarrays and RNA-seq to survey the intracellular environment, revealing thousands of genes that respond to IFN-α (84). However, our results propose a more complex scenario in which a limited set of genes display a robust increase both at the RNA and protein levels, while many others show changes in RNA that are not matched at the protein level or *vice versa*. Interestingly, robustly expressed ISGs follow a defined pattern in which RNA expression is noticeable early upon IFN-α stimulation (here 4 hpt), while the transcriptional response consolidates into protein expression at later time points (here 20 hpt). Robustly expressed ISGs (both at mRNA and protein level) are strongly enriched in ISGs with proven capacity to suppress infection (58, 59), and are conserved in the IFN response across mammalian species (89).

Conversely, a large group of genes displayed changes at the RNA level that are not reflected at the protein level. Discordance between protein and RNA has been observed in different cellular conditions (94, 108, 109, 110, 111), and our work demonstrates that it also occurs during the response to IFN-α. These discordances have been observed across different experimental conditions and are not artifacts caused by differences in analytical methods (112). This must now be considered when interpreting experiments from IFN-treated cells using RNA-centric approaches. Inconsistencies between RNA and protein can be explained by the diverse array of regulatory processed that collectively define gene expression, including RNA transcription, translation, and turnover, as well as protein homeostasis (113, 114, 115). Conceptually, there are two possible biological explanations for protein/RNA discordance: (i) transcriptional noise that has limited influence in the proteome because of buffering mechanisms involving protein homeostasis that compensate for stochastic transcriptome fluctuations (116, 117, 118, 119, 120, 121). This is likely the case of IFN-α downregulated genes as their overlapping across transcriptomic analyses is sparse, which is compatible with transcriptional noise (89, 90). ii) The gap between mRNA and protein “clocks” caused by newly synthesized mRNAs requiring time to produce its encoded protein to a detectable level. It should be noted that this gap could cause the proteins associated with mRNAs induced late upon IFN-α treatment (*e.g.* 20 hpt) to be missed in the present analysis. Notably, several of these late responder genes are involved in apoptosis (supplemental Fig. S4*F*), which suggests the existence of a late wave of gene expression that may induce cell death as a last resort against infection, after very long exposure to IFN-α.

Transcriptomic analysis of IFN-α also reveals a population of transcripts that are downregulated and have been associated with a higher CpG content (122). However, we did not observe an effect of the loss of these mRNAs at the proteome level at 20 hpt, suggesting the existence of mechanisms to maintain the homeostat of their encoded proteins. Conversely, 6 proteins had lower abundance after IFN-α treatment, while the levels of their cognate mRNAs remained unaltered. While these cases are small, our data suggests the existence of mechanisms that regulate protein stability, perhaps through ubiquitination pathways (123).

The intracellular environment can also be regulated by subtle mechanisms that do not require changes in protein levels, for example, *via* post-translational modifications (PTMs). Our data revealed changes in the phosphorylation status of hundreds of proteins as early as 10 min after interferon treatment. The importance of “rapid responses” is illustrated in supplemental Fig. S2, *A*–*C*, which shows that short IFN-α treatments cause a substantial inhibition of infection. Conversely, HEK293T cells displayed reduced capacity to suppress virus infection upon short IFN-α treatment (supplemental Fig. S2*A*). This suggests that HEK293 and HEK293T cells may also differ in their ability to mediate rapid responses to infection. However, if this phenomenon is due to differential activation of signaling pathways and, consequently, the configuration of PTMs, or to the abundance of the modified proteins requires further investigation. Interestingly, many of these phosphorylation events involve RBPs, which are central players in virus infection, promoting and restricting infection (103, 124). Many of these phosphosites are placed near or within the RBDs of these RBPs. Given the negative charge of the phosphorylated amino acid and the highly negative nature of the phosphate backbone of RNA, these post-translational modifications are expected to impair the ability of RBPs to bind RNA. However, it is difficult to distinguish between spurious and functionally relevant phosphosites, and further research should focus on characterizing their functional impact individually.

Through integrated omics analysis, we identified a group of cellular proteins with no or poorly characterized antiviral roles. Our assays revealed virus-inhibitory effects for most proteins tested (13/15 for SINV and 6/9 for HIV-1), ranging from very mild to strong. It is worth noting that two candidate genes, HNRNPD and POLDIP3, were selected based on their IFN-α responses at the phosphorylation level. These two proteins have been reported to bind to the RNA of multiple types of viruses and have multifaceted roles in regulating virus replications (125, 126, 127, 128, 129). Our assays confirmed their inhibitory effects against SINV and HIV-1, but whether the IFN-α induced phosphorylation of these proteins is important in the infection phenotypes must be further investigated. Our results with SINV infection also revealed different inhibitory mechanisms, with some proteins targeting the early stages of virus infection (*e.g.* UBE2L6), and others participating throughout the course of infection (e.g. EPSTI1). The mechanism underlying these different kinetics of antiviral effects remains an interesting topic for future studies.

Our data provide a rich resource for studying determinants of virus permissiveness across cell states, and IFN-α response across transcriptome, proteome, and phosphoproteome. The proteome-wide differential expressions between HEK293 and more permissive HEK293 T cells highlight the need to carefully distinguish these 2 cell lines in studies in virology, immunity, and related fields. The multi-omic analysis shows that transcriptome variations cannot fully explain the observed proteome changes, calling for careful consideration and validation.

## Data Availability

The mass spectrometry proteomics data have been deposited to the ProteomeXchange Consortium *via* the PRIDE (130) partner repository with the accession number PXD052223.

The RNA-seq data have been deposited to the Gene Expression Omnibus (GEO) repository with assession number GSE267610.

## Supplemental data

This article contains supplemental data.

## Conflict of interest

The authors declare that they have no conflicts of interest with the contents of this article.
