# Supplementary material for: Omics Analyses Uncover Host Networks Defining Virus-Permissive and -Hostile Cellular States
Source: Mol Cell Proteomics. 2025 Apr 7;24(5):100966. doi: 10.1016/j.mcpro.2025.100966 (PMC12136899; doi:10.1016/j.mcpro.2025.100966)
Supplement: Figure S1 [file mmc1.pdf]

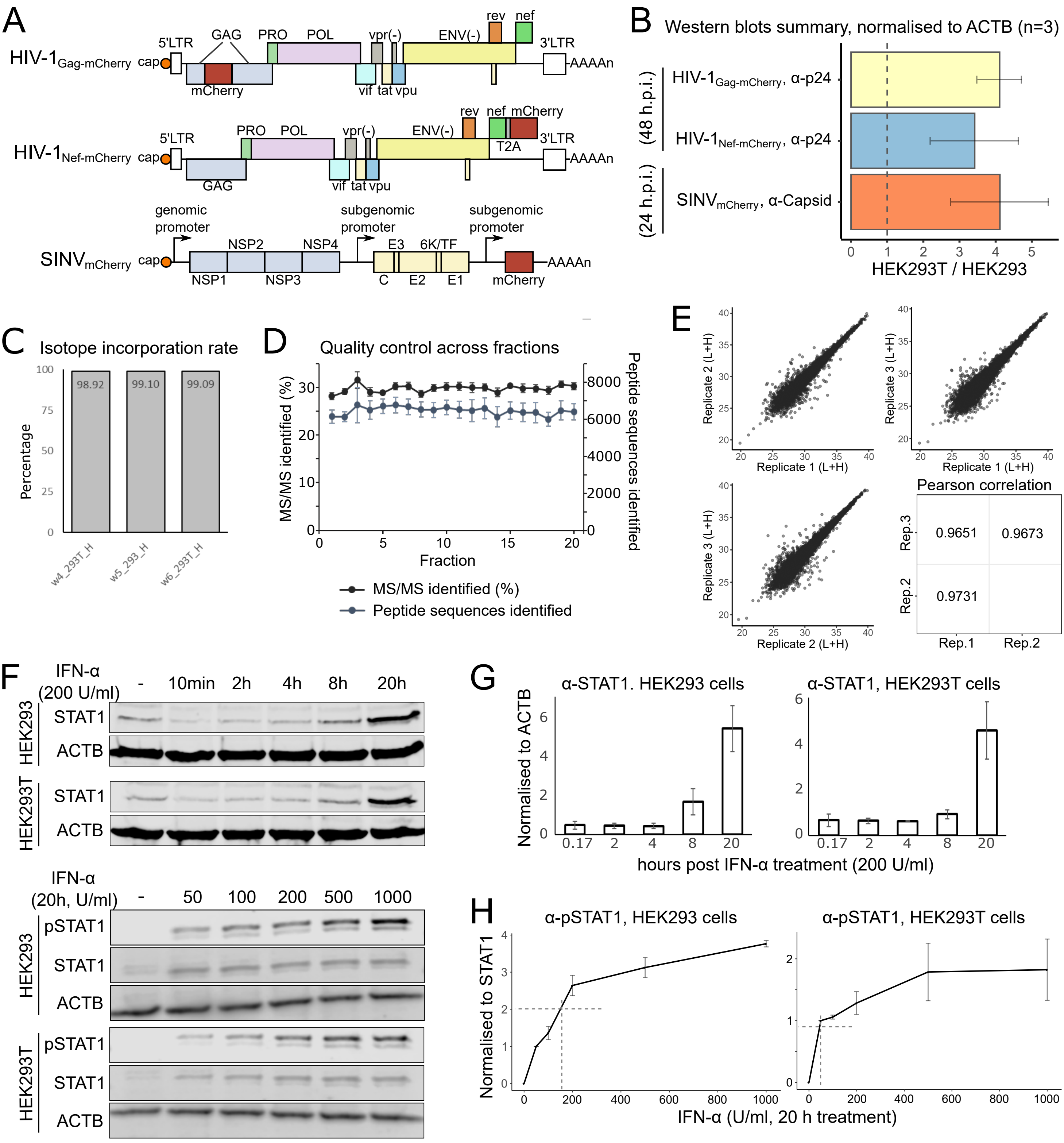

I

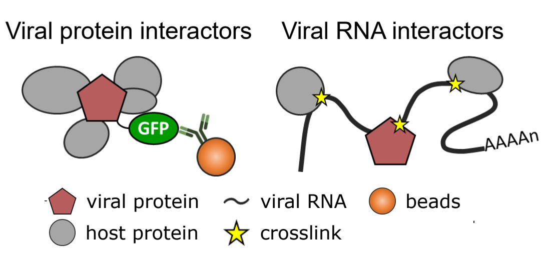

Viral protein interactors

Publication	No. of viral proteins	No. of identified interactors
Pichlmair, et.al., 2012	69	579
Jaeger, et.al., 2012	16	437
Gorden, et.al., 2020	27	332
Shah, et.al., 2018	22	632

Viral RNA interactors

Publication	No. of virus species	No. of identified interactors
Iselin, et.al., 2022 (review)	11	195
