# Supplementary figures and images for: Omics Analyses Uncover Host Networks Defining Virus-Permissive and -Hostile Cellular States
Source: Mol Cell Proteomics. 2025 Apr 7;24(5):100966. doi: 10.1016/j.mcpro.2025.100966 (PMC12136899; doi:10.1016/j.mcpro.2025.100966)

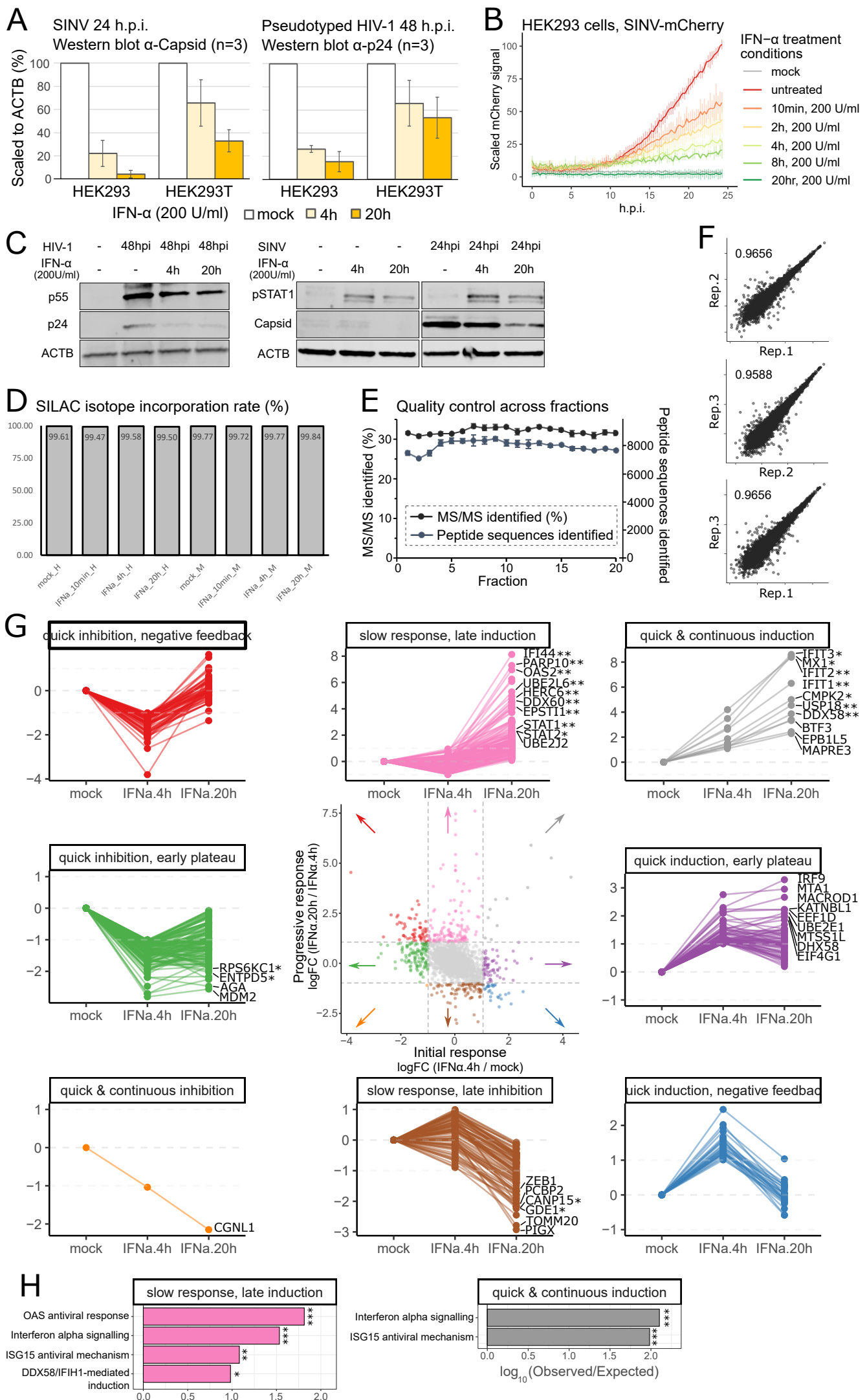

Supplement: Figure S2 [file mmc2.pdf]

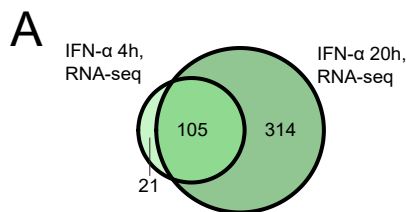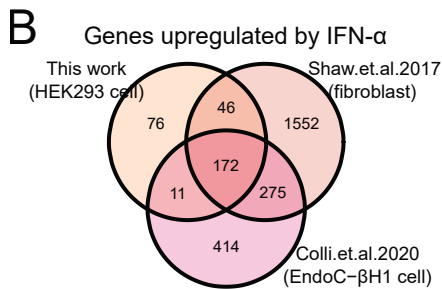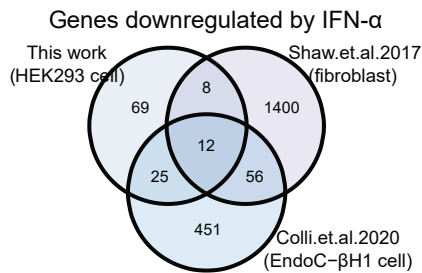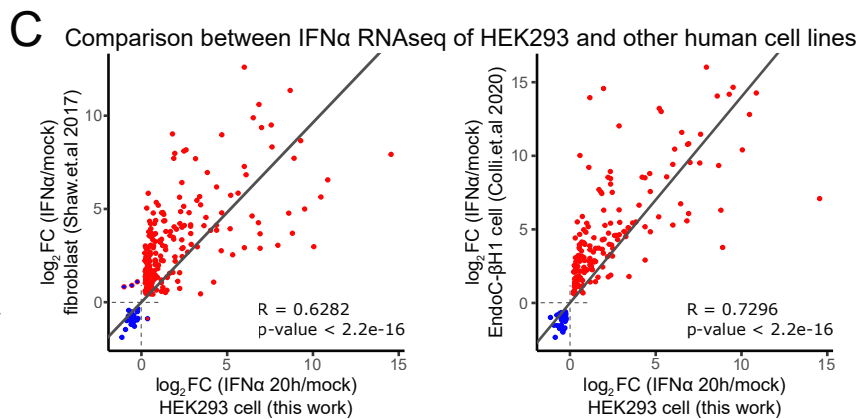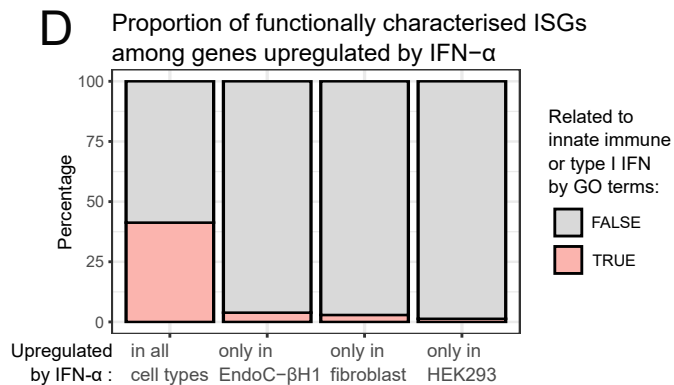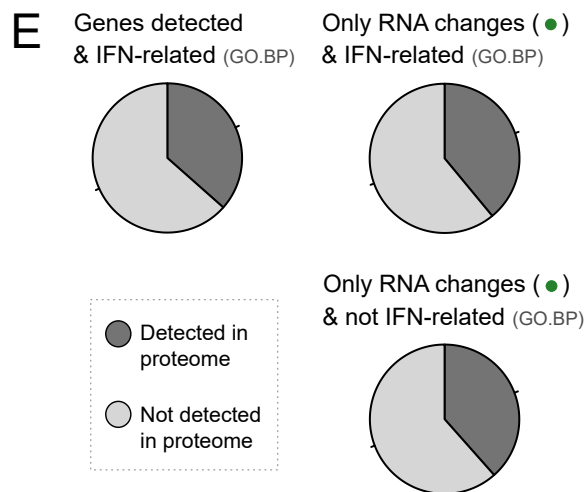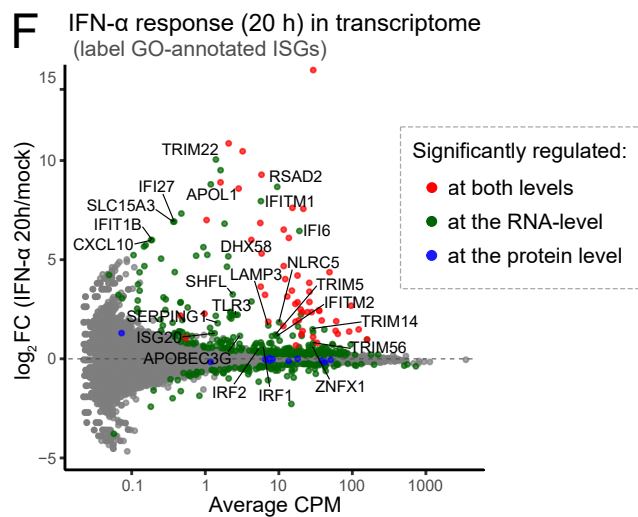

Supplement: Figure S3 [file mmc3.pdf]

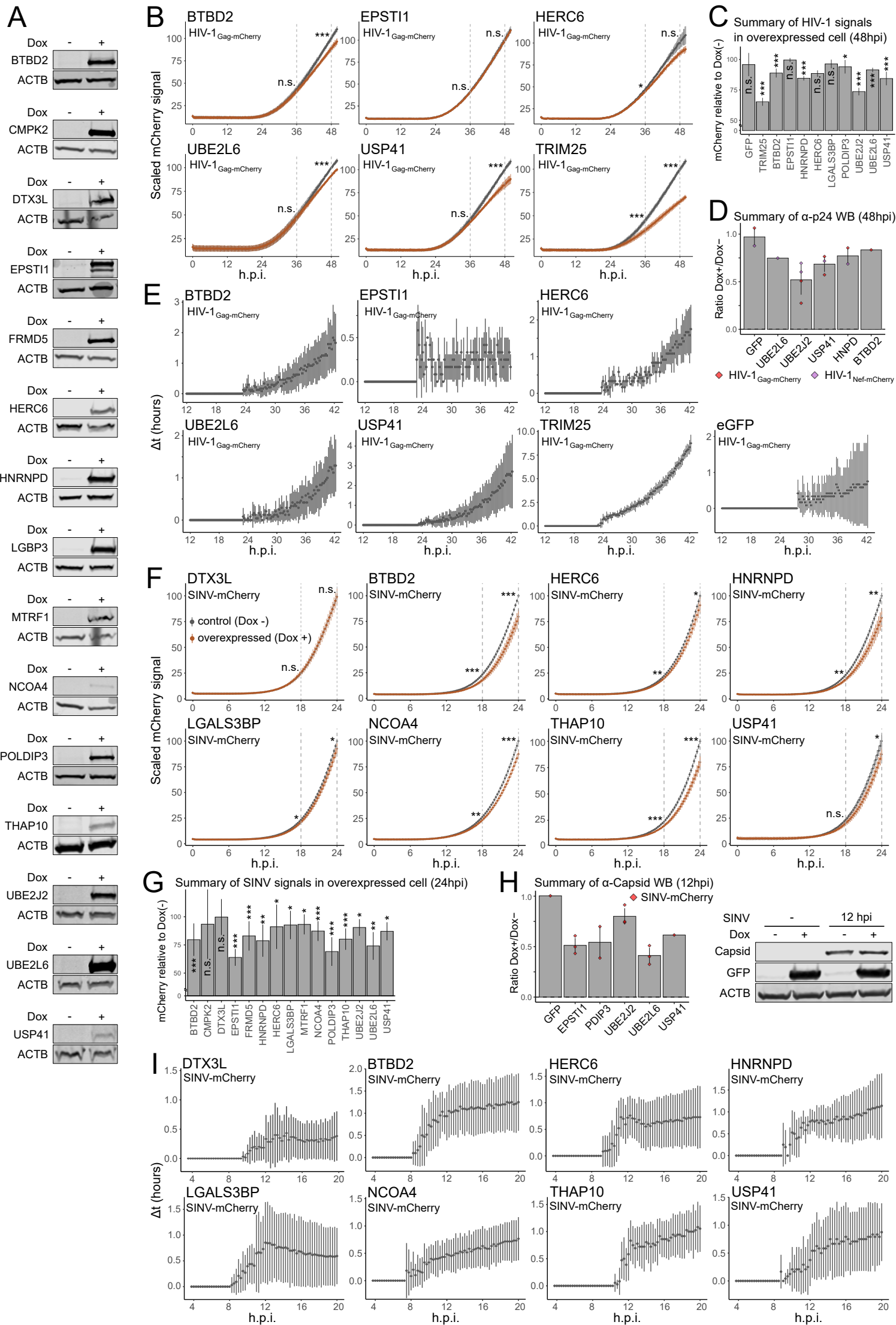

Supplement: Figure S6 [file mmc6.pdf]
