# Supplementary material for: Omics Analyses Uncover Host Networks Defining Virus-Permissive and -Hostile Cellular States
Source: Mol Cell Proteomics. 2025 Apr 7;24(5):100966. doi: 10.1016/j.mcpro.2025.100966 (PMC12136899; doi:10.1016/j.mcpro.2025.100966)
Supplement: Figure S4 [file mmc4.pdf]

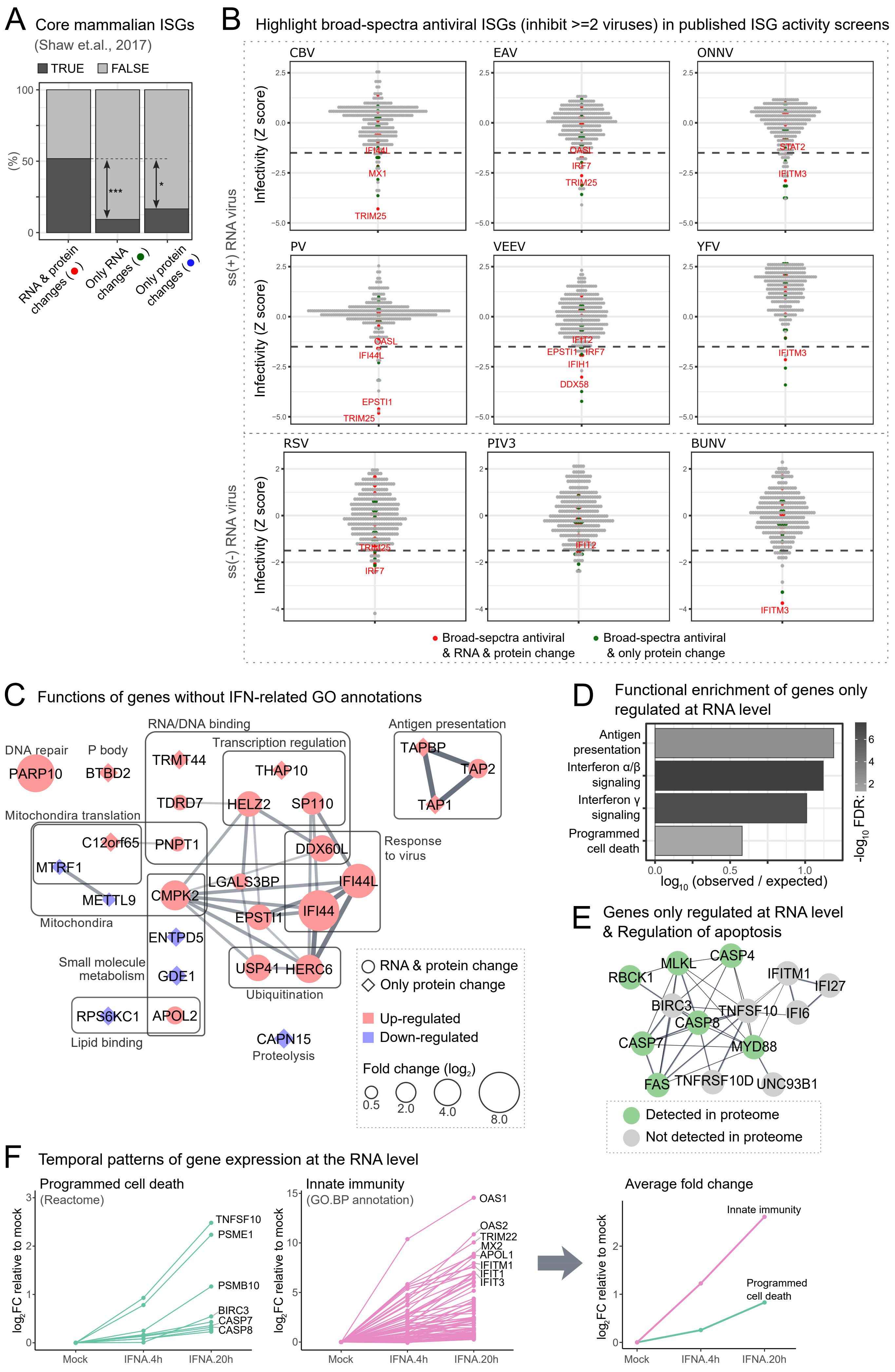
A
Core mammalian ISGs
(Shaw et.al., 2017)
TRUE
FALSE
(%)
100
50
0
***
*
RNA & protein changes
Only RNA changes
Only protein changes
B
Highlight broad-spectra antiviral ISGs (inhibit >=2 viruses) in published ISG activity screens
ss(+) RNA virus
ss(-) RNA virus
Infectivity (Z score)
CBV
EAV
ONNV
PV
VEEV
YFV
RSV
PIV3
BUNV
IFI44L
MX1
TRIM25
OASL
IRF7
STAT2
IFITM3
EPSTI1
IFIT2
IFIH1
DDX58
IFIT2
Broad-sepctra antiviral & RNA & protein change
Broad-spectra antiviral & only protein change
C
Functions of genes without IFN-related GO annotations
DNA repair
P body
RNA/DNA binding
Transcription regulation
Antigen presentation
PARP10
BTBD2
TRMT44
THAP10
TDRD7
HELZ2
SP110
TAPBP
TAP2
TAP1
Mitochondria translation
C12orf65
PNPT1
DDX60L
Response to virus
MTRF1
METTL9
Mitochondria
CMPK2
LGALS3BP
IFI44L
EPSTI1
IFI44
ENTPD5
Small molecule metabolism
GDE1
USP41
HERC6
Ubiquitination
RPS6KC1
APOL2
Lipid binding
CAPN15
Proteolysis
RNA & protein change
Only protein change
Up-regulated
Down-regulated
Fold change (log2)
0.5
2.0
4.0
8.0
D
Functional enrichment of genes only regulated at RNA level
Antigen presentation
Interferon α/β signaling
Interferon γ signaling
Programmed cell death
0.0
0.5
1.0
log10 (observed / expected)
-log10 FDR:
6
4
2
E
Genes only regulated at RNA level & Regulation of apoptosis
RBCK1
MLKL
CASP4
IFITM1
IFI27
BIRC3
TNFSF10
IFI6
CASP8
CASP7
MYD88
FAS
TNFRSF10D
UNC93B1
Detected in proteome
Not detected in proteome
F
Temporal patterns of gene expression at the RNA level
Programmed cell death
(Reactome)
log2FC relative to mock
TNFSF10
PSME1
PSMB10
BIRC3
CASP7
CASP8
Mock
IFNA.4h
IFNA.20h
Innate immunity
(GO.BP annotation)
OAS1
OAS2
TRIM22
MX2
APOL1
IFITM1
IFIT1
IFIT3
Average fold change
Innate immunity
Programmed cell death
