# Supplementary material for: Omics Analyses Uncover Host Networks Defining Virus-Permissive and -Hostile Cellular States
Source: Mol Cell Proteomics. 2025 Apr 7;24(5):100966. doi: 10.1016/j.mcpro.2025.100966 (PMC12136899; doi:10.1016/j.mcpro.2025.100966)
Supplement: Figure S5 [file mmc5.pdf]

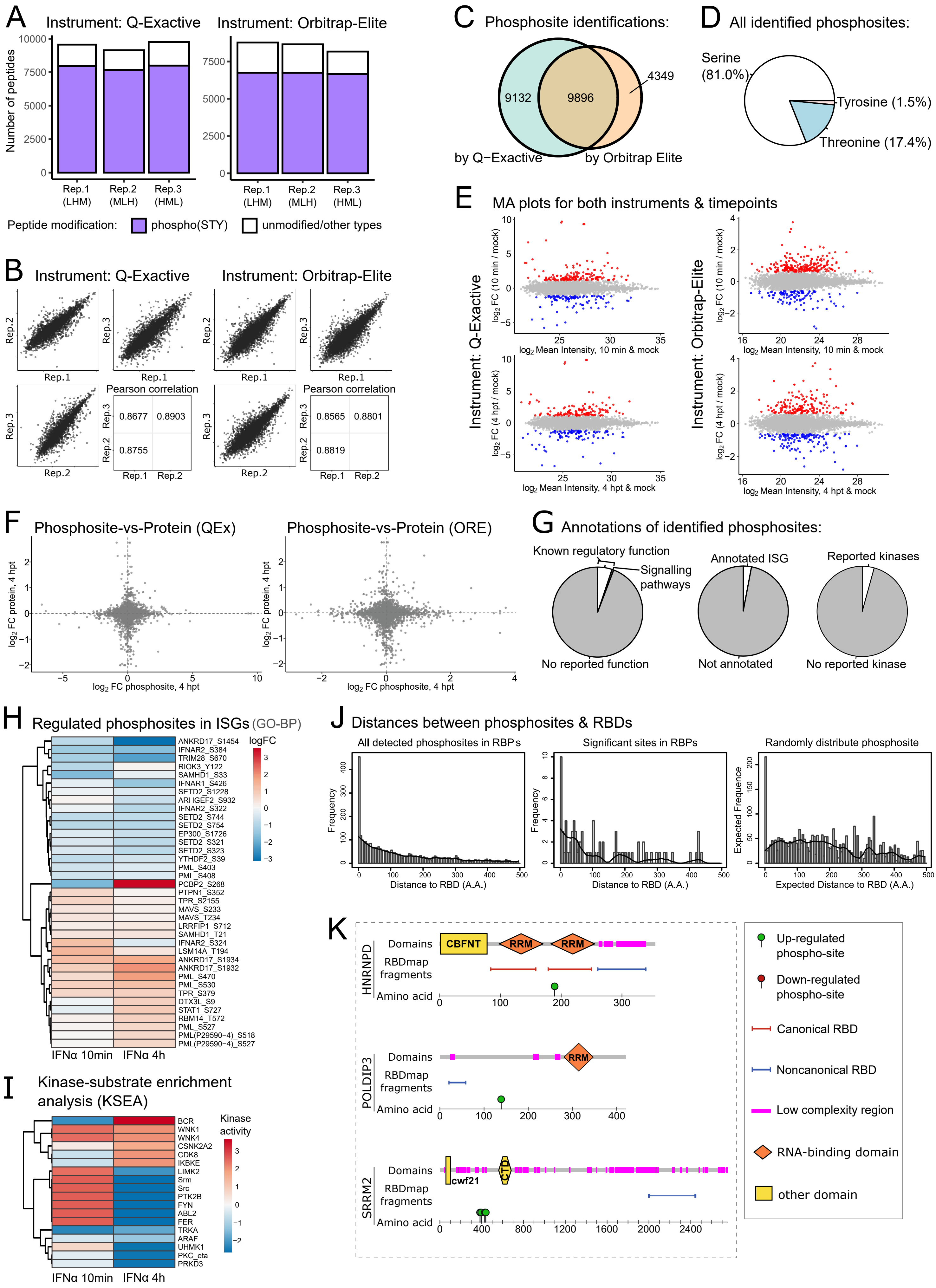
A
Instrument: Q-Exactive
Instrument: Orbitrap-Elite
Number of peptides
Rep.1 (LHM)
Rep.2 (MLH)
Rep.3 (HML)
Peptide modification: phospho(STY) unmodified/other types
B
Instrument: Q-Exactive
Instrument: Orbitrap-Elite
Pearson correlation
0.8677 0.8903
0.8755
0.8565 0.8801
0.8819
C
Phosphosite identifications:
9132
9896
4349
by Q−Exactive
by Orbitrap Elite
D
All identified phosphosites:
Serine (81.0%)
Tyrosine (1.5%)
Threonine (17.4%)
E
MA plots for both instruments & timepoints
Instrument: Q-Exactive
Instrument: Orbitrap-Elite
log2 FC (10 min / mock)
log2 FC (4 hpt / mock)
log2 Mean Intensity, 10 min & mock
log2 Mean Intensity, 4 hpt & mock
F
Phosphosite-vs-Protein (QEx)
Phosphosite-vs-Protein (ORE)
log2 FC protein, 4 hpt
log2 FC phosphosite, 4 hpt
G
Annotations of identified phosphosites:
Known regulatory function
Signalling pathways
No reported function
Annotated ISG
Not annotated
Reported kinases
No reported kinase
H
Regulated phosphosites in ISGs (GO-BP)
logFC
ANKRD17_S1454
IFNAR2_S384
TRIM28_S670
RIOK3_Y122
SAMHD1_S33
IFNAR1_S426
SETD2_S1228
ARHGEF2_S932
IFNAR2_S322
SETD2_S744
SETD2_S754
EP300_S1726
SETD2_S321
SETD2_S323
YTHDF2_S39
PML_S403
PML_S408
PCBP2_S268
PTPN1_S352
TPR_S2155
MAVS_S233
MAVS_T234
LRRFIP1_S712
SAMHD1_T21
IFNAR2_S324
LSM14A_T194
ANKRD17_S1934
ANKRD17_S1932
PML_S470
PML_S530
TPR_S379
DTX3L_S9
STAT1_S727
RBM14_T572
PML_S527
PML(P29590−4)_S518
PML(P29590−4)_S527
IFNα 10min
IFNα 4h
I
Kinase-substrate enrichment analysis (KSEA)
Kinase activity
BCR
WNK1
WNK4
CSNK2A2
CDK8
IKBKE
LIMK2
Srm
Src
PTK2B
FYN
ABL2
FER
TRKA
ARAF
UHMK1
PKC_eta
PRKD3
IFNα 10min
IFNα 4h
J
Distances between phosphosites & RBDs
All detected phosphosites in RBPs
Significant sites in RBPs
Randomly distribute phosphosite
Frequency
Expected Frequence
Distance to RBD (A.A.)
Expected Distance to RBD (A.A.)
K
HNRNPD
POLDIP3
SRRM2
Domains
RBDmap fragments
Amino acid
CBFNT
RRM
CTD
cwf21
Up-regulated phospho-site
Down-regulated phospho-site
Canonical RBD
Noncanonical RBD
Low complexity region
RNA-binding domain
other domain
