# Supplementary material for: Omics Analyses Uncover Host Networks Defining Virus-Permissive and -Hostile Cellular States
Source: Mol Cell Proteomics. 2025 Apr 7;24(5):100966. doi: 10.1016/j.mcpro.2025.100966 (PMC12136899; doi:10.1016/j.mcpro.2025.100966)
Supplement: Supplementary figure legends [file mmc13.docx]

Omics analyses uncover host networks defining virus-permissive and -hostile cellular states

Honglin Chen^1,2^, Philip D Charles^4^, Quan Gu^1^, Sabrina Liberatori^2^, David L Robertson^1^, Massimo Palmarini^1^, Sam J Wilson^5^, Shabaz Mohammed^2,3,6^* and Alfredo Castello^1^*

^1^ MRC-University of Glasgow Centre for Virus Research, Glasgow, Scotland (UK)

^2^ Department of Biochemistry, University of Oxford, South Parks Road, Oxford, UK.

^3^ The Rosalind Franklin Institute, Oxfordshire, UK

^4^ Big Data Institute, University of Oxford, Old Road Campus, UK

^5^ Cambridge Institute of Therapeutic Immunol & Infect Disease, Jeffrey Cheah Biomedical Centre, Cambridge Biomedical Campus, Puddicombe Way, UK

^6^ Department of Chemistry, University of Oxford, Mansfield Road, Oxford, UK

* Corresponding authors:

[alfredo.castello@glasgow.ac.uk](mailto:alfredo.castello@glasgow.ac.uk) (AC)

[shabaz.mohammed@chem.ox.ac.uk](mailto:shabaz.mohammed@chem.ox.ac.uk) (SM)

# **SUPPLEMENTARY MATERIALS**

**Supplementary Figure S1**: Host processes underlying different permissiveness between HEK293 and HEK293T cells

**Supplementary Figure S2**: IFN-α stimulation induce temporal changes in HEK293 cell proteome

**Supplementary Figure S3**: IFN-α stimulation induces signature antiviral response in HEK293 cell transcriptome and discordant mRNA-protein changes

**Supplementary Figure S4**: Antiviral activities and other processes among IFN-α regulated genes in HEK293 cells

**Supplementary Figure S5**: Phosphoproteome analysis of HEK293 cells after IFN-α stimulation

**Supplementary Figure S6**: Virus-inhibitory effects against SINV and HIV-1 in candidate genes

**Supplementary Table S1**: HEK293 vs HEK293T deep proteome results

**Supplementary Table S2**: IFN-α vs mock deep proteome results

**Supplementary Table S3**: IFN-α vs mock RNA-sequencing results

**Supplementary Table S4**: IFN-α vs mock deep phosphoproteome results

**Supplementary Table S5**: Kinase-substrate enrichment analysis results

**Supplementary Table S6**: Candidate genes for stable expression cell line

## **SUPPLEMENTARY FIGURE LEGENDS**

**Figure S1. Host processes underlying different permissiveness between HEK293 and HEK293T cells**

(A) Diagrams of HIV-1 genome with mCherry insertion in-frame with Gag (HIV-1Gag-mCherry, top) or Nef (HIV-1Nef-mCherry, mid), and SINV genome with mCherry insertion (SINVmCherry, bottom). (B) Summary of Western blot experiments comparing viral capsid abundance between HEK293 and HEK293T cells (n=3). (C) SILAC isotope incorporation rates assessed by LC-MSMS. (D) MS/MS identification rates and numbers of peptide sequences identified in each fraction were drawn in line plot for quality control. Error bars represent standard deviation from three replicates. (E) Scatter plots comparing log-transformed intensities between replicates, Pearson correlation coefficients for each contrast pair were shown in corresponding blocks (S1 Table). (F) Western blotting with antibodies of ACTB, STAT1, and STAT1-pTyr701 (pSTAT1) from IFN-α treated HEK293 and HEK293T cells. Cells were treated with 200 U/ml of IFN-α for different periods of time (top) or with different dosages of IFN-α for 20 hours (bottom). (G) Barplot summarising ACTB-normalised STAT1 signals from triplicate Western blots as in Fig S1F (top). (H) Barplot summarising STAT1-pTyr701 signals normalised to STAT1 from triplicate Western blots as in Fig S1F (bottom). (I) Schematic of viral protein/RNA interactors (grey) included in enrichment analysis (left), with tables summarising publications and numbers of interactors determined in each (right).

**Figure S2. IFN-α stimulation induce temporal changes in HEK293 cell proteome**

(A) Barplot summarising ACTB-normalised viral capsid protein signals from triplicate Western blots as in Fig 2F. (B) Infection fitness of SINVmCherry in HEK293 cells treated with 200 U/ml of IFN-α for different periods of time. Fluorescence signals were measured every 15 min in culture condition using a plate reader (top, n=3). (C) Western blottings with antibodies of pSTAT1 and viral capsid proteins. Cells were infected at MOI=1 with HIV-1Nef-mCherry or SINVmCherry. (D) SILAC isotope incorporation rates assessed by LC-MSMS for IFN-α/mock proteome samples. (E) MS/MS identification rates and numbers of peptide sequences identified in each fraction were drawn in line plot for quality control for IFN-α/mock proteome samples. Error bars represent standard deviation from three replicates. (F) Scatter plots comparing log-transformed intensities between replicates for IFN-α/mock proteome samples. Pearson correlation coefficients for each contrast pair were shown in corresponding blocks (Table S2). (G) Scatter plot (centre) comparing log2 fold changes of IFN-α induced initial responses (4 hpt/mock) against progressive responses (20 hpt/4 hpt) for each protein. Temporal changes were divided into 8 groups by cut-off lines at log2 fold change of 1 and visualised in parallel coordinate plots. Top fold changes in each group were text labelled, significant changes were * labelled (*: FDR<0.1, **: FDR<0.01). (H) Enrichment analysis of Reactome pathways in groups in (G), P-values of enrichment were labelled in * (*: p<0.05, **: p<0.01, ***: p<0.001).

**Figure S3. IFN-α stimulation induces signature antiviral response in HEK293 cell transcriptome and discordant mRNA-protein changes**

(A) Venn diagrams comparing numbers of IFN-α induced significant changes at 4 hpt and 20 hpt at the RNA level (Table S3). (B) Venn diagrams of RNA-seq results of IFN-α response in different cell lines. Number of genes upregulated (top) or downregulated (bottom) in each cell line and respective publications were compared. (C) Scatter plots comparing log2 fold changes in RNA-seq results between different cell lines. Genes up-/down-regulated in HEK293 cells were filled with red/blue, and genes up-/down-regulated in other cell types were outlined with red/blue. (D) Proportions of GO-BP annotated ISGs among IFN-α upregulated genes identified in different cell lines. (E) Proportion of genes that were detected in proteome among total detected genes, and genes that only showed RNA-level response in RNA-seq. (F) MA plot of IFN-α induced transcriptome change at 20 hpt, with colour labels based on RNA-/protein-levels changes and text labels of GO-BP annotated ISGs.

**Figure S4. Antiviral activities and other processes among IFN-α regulated genes in HEK293 cells**

(A) Proportions of core mammalian ISGs among genes regulated by IFN-α in HEK293 cells at RNA/protein levels. (B) Dot plots of FACS screening results listed in Fig 4B. Genes with broad-spectrum antiviral activities were colour labelled based on IFN-α response at RNA/protein levels in HEK293 cells. ISGs with Z < -1.5 for each virus were text labelled. (C) Protein-protein interaction network of genes that have protein-level IFN-α response and have no IFN-related GO-BP annotation. (D) Enrichment analysis of Reactome pathways for genes that showed RNA-level, but no protein-level IFN-α response. (E) Protein-protein interaction network of genes involved in regulation of apoptosis, and showed RNA-level, but no protein-level IFN-α response. Genes detected in proteome dataset but determined not significant were green coloured. (F) Parallel coordinate plots showing mRNA expression patterns for genes involved in programmed cell death (left) or innate immunity (mid). Only genes with FDR < 0.05 in RNA-seq result were plotted. Overall expression patterns of two processes were compared by respective average fold change (right).

**Figure S5. Phosphoproteome analysis of HEK293 cells after IFN-α stimulation**

(A) Barplots showing numbers of phosphorylated and non-phosphorylated peptides identified in each SILAC sample from two instrument configurations. (B) Scatter plots comparing log-transformed intensities between replicates, with Pearson correlation coefficients for respective contrast pair. Results from two instrument configurations were displayed separately. (C) Venn diagram comparing numbers of phosphosites identified using two instrument configurations. (D) Proportions of serine/threonine/tyrosine phosphorylation identified in combined result. (E) MA plots of IFN-α induced phosphoproteomic changes at 10 min and 4 hpt obtained with two instrument configurations: Orbitrap-Elite (ORE; left) and Q-Exactive (QEx; right). Phosphosites with FDR < 0.1 in either moderated T statistic or Z statistic were colour labelled and determined as sites of significant changes (Table S4). (F) Scatter plots comparing IFN-α induced changes at proteome and phosphoproteome levels at 4 hpt. (G) Proportions of functional annotations among all identified phosphosites (left); IFN-related GO-BP annotations among all identified phosphoproteins (mid); kinase annotations among all identified phosphosites (right). (H) Heatmap of IFN-α regulated phosphosites within proteins that have IFN-related GO-BP annotations. (I) Heatmap of kinase-substrate enrichment analysis (KSEA) result (Table S5). (J) Histograms of phosphosites distribution base on their distances (number of amino acids) relative to RBDs. (K) Schematics of RBPs that contains IFN-α regulated phosphosites. “Domains” showed annotations from SMART and Pfam; “RBDmap fragment” showed RNA-interacting peptide fragments identified in proteomics-based interactome analysis (69).

**Figure S6. Virus-inhibitory effects against SINV and HIV-1 in candidate genes**

(A) Expression candidate genes with eGFP fusion in inducible stable lines verified with Western blottings using antibodies of eGFP and ACTB. (B) Infection fitness of HIV-1Gag-mCherry in HEK293 inducible stable lines expressing candidate genes with eGFP fusion. Fluorescence signals were measured every 15 min in culture condition using a plate reader (top, n=3). (C) Summary of plate reader results for HIV-1Gag-mCherry assays as in Fig S6B at 48 hpi. (D) Summary of Western blot experiments that assess virus-inhibitory effects of candidates using HIV-1. Numbers of replicates and type of HIV-1 replicon used were shown in red (HIV-1Gag-mCherry) and purple (HIV-1Nef-mCherry) dots. (E) Delay analysis from 12 to 42 hpi for HIV-1 infected stable lines overexpressing other candidate genes. (F) Infection fitness of SINVmCherry in HEK293 inducible stable lines expressing candidate genes with eGFP fusion. Fluorescence signals were measured every 15 min in culture condition using a plate reader (top, n=3). (G) As in Fig S6C but for assays with SINVmCherry at 24 hpi. (H) As in Fig S6D but for SINVmCherry. (I) As in Fig S6E but for SINV infected cells ranging from 4 to 20 hpi.
